# Medium- and long-chain triacylglycerols and di-unsaturated fatty acyl-palmitoyl-glycerols in Chinese human milk: Association with region during the lactation

**DOI:** 10.3389/fnut.2022.1040321

**Published:** 2022-10-14

**Authors:** Jiahui Yu, Zhiyuan Yan, Lijuan Mi, Lei Wang, Zhengdong Liu, Xingwang Ye, QingZhe Jin, Jinzhu Pang, Wei Wei, Xingguo Wang

**Affiliations:** ^1^Collaborative Innovation Center of Food Safety and Quality Control in Jiangsu Province, School of Food Science and Technology, Jiangnan University, Wuxi, China; ^2^Inner Mongolia Mengniu Dairy (Group) Co., Ltd., Beijing, China; ^3^Yashili International Group Co., Ltd., Guangzhou, China

**Keywords:** human milk fat, triacylglycerol, medium-and long-chain triacylglycerols, di-unsaturated fatty acyl-palmitoyl-glycerols, lactation stage

## Abstract

The triacylglycerols (TAGs) of medium- and long-chain triacylglycerols (MLCT) and di-unsaturated fatty acyl-palmitoyl-glycerols (UPU) in human milk provide better nutritional effects, and should be prioritized as crucial focuses on neonatal nutrition research. However, little has been done on the influences of the lactation stage and regional diversity on MLCT and UPU. In this study, we collected 204 human milk samples during colostrum, 1st and 4th month from the north (Baotou), central (Beijing), east (Jinan), southwest (Kunming), southeast (Shenzhen), and northwest (Xining) regions of China. There were 122 species of TAGs detected with UPLC-Q-TOF-MS, including 60 kinds of MLCT and 15 kinds of UPU. The MLCT and UPU type TAGs in human milk were ~27 and ~38%, respectively. The sum content of MLCT and UPU in human milk was stable. Compared to the regional diversity, lactation stages showed more obvious influences on MLCT and UPU composition. Moreover, a summary of TAG studies indicated that Chinese human milk showed a higher ratio of O-P-L to O-P-O than in western countries.

## Introduction

Human milk provides the optimum diet for newborns. The complex constituents, such as lipids, protein, carbohydrates, and other micronutrients, are appropriate for the physiological immaturity of infants (1). Human milk fat constitutes 3–5% of human milk, of which the majority are triacylglycerols (TAGs, 98–99%) (2). Human milk TAGs provide infants with the required energy, essential fatty acids, and other bioactive lipids (3). One TAG molecule is composed of a three-carbon glycerol backbone esterified with three fatty acids. Based on the chain length and saturation degree of fatty acids, different TAG structures differ in metabolism rate.

Medium- and long-chain triacylglycerol (MLCT) contains both medium-chain fatty acid (MCFA, C_8_-C_14_) and long-chain fatty acid. MLCT facilitates better digestion and absorption of lipids to provide rapid energy than long-chain triacylglycerol (LCT), contributed by the direct absorption of MCFA. Moreover, MLCT compensates for the drawbacks of medium-chain triacylglycerol (MCT) by the incorporation of long-chain fatty acid (4, 5). Human milk contains 7–23% MCFA of the total fatty acids and about 30% MLCT of the TAG molecules (5). Especially, human milk was abundant in TAG with one MCFA and two long-chain fatty acids (MLL type, 20.89–31.47%), while infant formulas were rich in TAG with three MCFAs (MMM type) or two MCFAs and one long-chain fatty acid (MML type) (6). The significant gap urged the priority of MLCT on neonatal nutrition research.

Based on the saturation degree, it was reported that 16:0 on the *sn*-2 position could increase weight gain and bone mineral content and lower total stool fatty acid soaps (7). In human milk, 59% TAGs contained 16:0, and 32% TAGs were di-unsaturated fatty acyl-palmitoyl-glycerols (UPU) (8). However, the majority of studies made efforts on the benefit of abundant species in human milk, such as O-P-O (10%) or O-P-L (13%), neglecting other TAGs containing palmitic acid (8, 9). For example, L-P-L, O-P-La, and O-P-P are also presented in human milk with content more than 2% (8). Taking consideration of a category of TAGs, not a specific TAG, benefits a better understanding of the nutritional requirements of infants.

It was reported that human milk fat could be modified by multiple factors, such as lactation stages, regions, and genes (10, 11). The dynamic change of fat during lactation stages reflects infants' requirements. The vast territory of China leads to an abundant diet, which could influence the lipids in human milk. However, previous studies researched the influence of the lactation stage and regional diversity on the fatty acid composition, and rare studies reported the relationship to TAG composition (12, 13).

The unique structures of MLCT and UPU provide better nutritional effects and should be prioritized as crucial focuses in neonatal nutrition research. We hypothesized that the lactation stage and regional diversity influenced the MLCT and UPU characteristics in human milk. Therefore, we collected 204 human milk samples during colostrum, 1st and 4th months from the north (Baotou), central (Beijing), east (Jinan), southwest (Kunming), southeast (Shenzhen), and northwest (Xining) regions of China. This study benefits a better understanding of the nutritional requirements of infants from the perspective of TAGs.

## Materials and methods

### Reagents and standards

TAG standard mixture (8:0 TAG, 10:0 TAG, 12:0 TAG, 14:0 TAG and 16:0 TAG, 17811-1AMP) was obtained from Sigma-Aldrich Chemicals (Shanghai, China). HPLC grade of *n*-hexane (>99.9% purity) was bought from J&K Scientific, Ltd. (Beijing, China). Ammonia solution, ethanol, diethyl ether, and petroleum ether were obtained from Sinopharm Chemical Reagent Co. Ltd. (Shanghai, China).

### Human milk sample collection

Human milk samples were obtained from the north (Baotou), central (Beijing), east (Jinan), southwest (Kunming), southeast (Shenzhen), and northwest (Xining) regions of China to evaluate human milk TAGs composition. The Ethics Committee approved this study by Capital Pediatric Research Institute, Beijing (SHERLL2013007). All mothers were in healthy condition with full-term infants and signed informed consent forms.

The detailed information is shown in Table 1. This study covered 204 human milk samples obtained from 6 cities in China, including Baotou (*n* = 40), Beijing (*n* = 41), Jinan (*n* = 20), Kunming (*n* = 33), Shenzhen (*n* = 21), and Xining (*n* = 49). The maternal age ranged from 18 to 36 years old. Based on the lactation stages, the milk samples included colostrum (*n* = 28), 1st month (*n* = 67) and 4th month (*n* = 109). The inclusion criteria were lactating mothers with healthy infants, physically healthy, non-smoking and non-alcoholic consumers and signed informed consent forms. The collected samples were frozen and transported to the lab, and stored at −80°C until further analysis.

**Table 1:** Basic information of human milk samples collected from six cities in China.

	**Baotou**	**Beijing**	**Jinan**	**Xining**	**Kunming**	**Shenzhen**
Sample size						
Colostrum (*n* = 28)	0	7	0	10	11	0
1st month (*n* = 67)	20	17	0	19	11	0
4th month (*n* = 109)	20	17	20	20	11	21
Age at delivery (y)						
Colostrum		25.9 ± 2.6		28.0 ± 5.1	28.1 ± 2.4	
1st month	28.0 ± 3.1	26.8 ± 2.7		27.5 ± 4.3	28.1 ± 2.4	
4th month	28.1 ± 3.0	26.8 ± 2.7	28.9 ± 2.3	27.3 ± 4.2	28.1 ± 2.4	29.7 ± 2.3
Infant birth weight (kg)	3.4 ± 0.3	3.5 ± 0.4	3.4 ± 0.4	3.0 ± 0.4	3.1 ± 0.5	3.3 ± 0.4
Male (%)	65	75.6	50.0	38.8	70.9	35

### Total lipid extraction from human milk

The Röse-Gottlieb method was used to extract the total lipids in human milk, with some modifications (14). Briefly, 1 mL of ammonium hydroxide was mixed with 5 mL of human milk and heated at 65 ± 5°C for 20 min. After cooling to room temperature, 5 mL of ethanol, 10 mL of diethyl ether, and 10 mL of petroleum ether were used to extract lipids. Then, the upper phase was collected. The lower layer was extracted again with half the volume of solvents.

### TAG analysis using UPLC-Q-TOF-MS

The human milk fat was dissolved in *n*-hexane at a concentration of 0.3 mg/mL. Ultra-performance liquid chromatography coupled with quadrupole time-of-flight mass spectrometry (UPLC-Q-TOF-MS) (Waters, Milford, MA, USA) was used to detect TAGs following our previous studies (15). A UPLC BEH C18 (i.d. 2.1 × 50 mm × 1.9 μm) column was used. The mobile phase A was acetonitrile/isopropanol (1:9, v/v), and B was acetonitrile/water (4:6, v/v). Both the two mobile phases contained 10 mmol/L of ammonium. The mass spectrometry conditions: the capillary voltage was 3.5 kV, and cone voltage was 30 V; the ion source temperature was 100°C, and the desolvation temperature was 400°C; the desolvation gas and cone gas flow rate were 50 and 700 L/h, respectively. The mass range was from 200 to 1,500 m/z. TAGs were identified by Waters MassLynx software (version 4.1, Waters) and quantified by QI software (Waters, Milford, MA, USA). The quasi-molecular ions (M+NH4)^+^ was used to identify TAG molecular species, and the corresponding (DAG)^+^ fragment was found from the MS/MS spectrum. The accurate mass of (M+H-DAG)^+^ could be used to deduce the fatty acid. We did not differentiate TAG regioisomers and used the sign “-” to connect three fatty acids to represent a TAG molecular, like O-P-O.

### Statistical analysis

Results were performed in triplicate and reported as mean ± standard deviation. Box-plots were carried out with Origin (version 9.0, USA), and orthogonal partial least squares discrimination analysis (OPLS-DA) was carried out using Umetrics SIMCA (version 14.1, Umea, Sweden). The variable importance projection (VIP) scores plot was analyzed using an online tool (https://www.metaboanalyst.ca/home.xhtml). The differences of TAGs were analyzed using a one-way analysis of variance (ANOVA) and Duncan post-tests using IBM SPSS Statistics (version 20.0, New York, USA).

## Results

### Identified TAGs in six cities

A total of 122 kinds of TAGs were identified and quantified using UPLC-Q-TOF-MS combined with Masslynx and QI software for processing data (Supplementary Table 1). The acyl carbon number of human milk TAGs varied from 36 to 56, and the number of double bonds ranged from 0 to 7. The most abundant TAG in human milk was O-P-L (16.04 ± 2.32%), followed by O-P-O (13.90 ± 2.52%) and L-L-P (5.25 ± 1.76%). The contents of S-O-P, O-O-L, S-L-P, O-P-P and O-P-La were over 3%.

**Figure 1:**
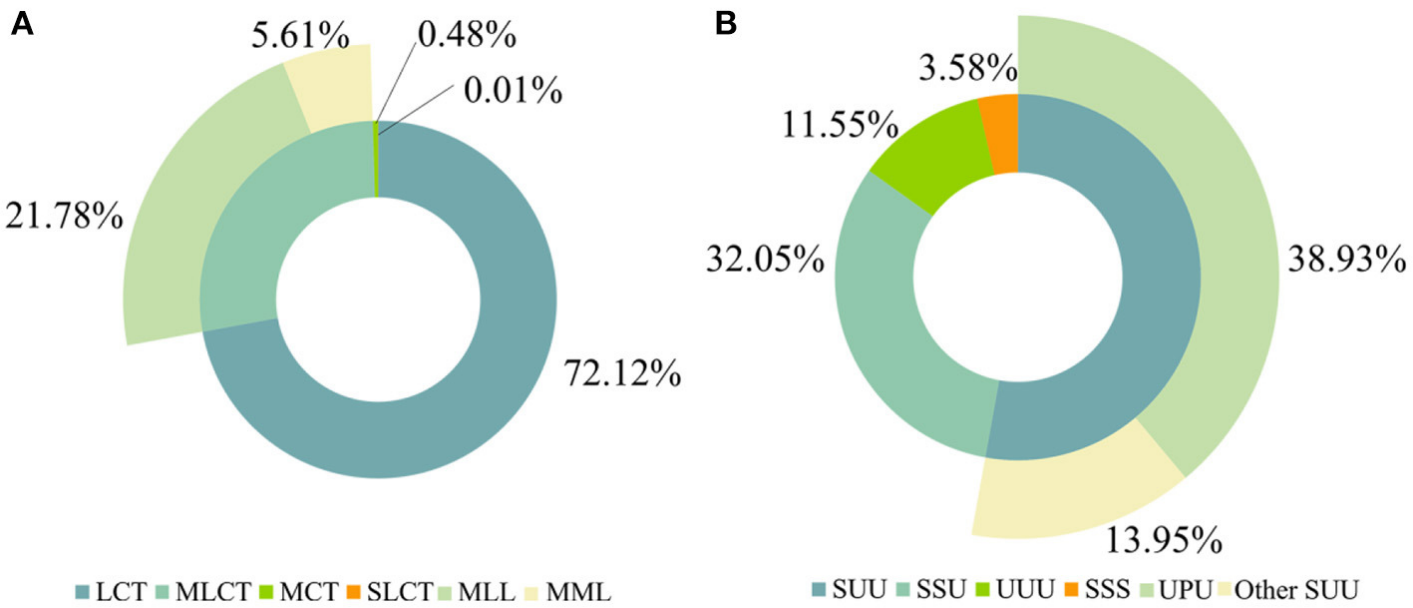
Composition characteristics of TAGs in human milk based on fatty acid chain length **(A)** and fatty acid saturation **(B)**. S, short-chain fatty acids; M, medium-chain fatty acids; L, long-chain fatty acids; S, saturated fatty acids; U, unsaturated fatty acids; P, 16:0.

Based on the fatty acid chain length, TAGs can be classified into four kinds: short- and long-chain TAGs (SLCT), MCT, MLCT, and LCT. Figure 1A shows the composition of TAGs in human milk based on fatty acid chain length. The majority TAG species in human milk was LCT (72.12%), followed by MLCT (27.39%), MCT (0.48%), and SLCT (0.01%). MLCT was constituted by MLL-type TAGs (21.78%) and MML-type TAGs (5.61%).

Based on the saturation degree of fatty acids, TAGs can be divided into four groups: TAGs esterified with three saturated fatty acids (SSS), TAGs esterified with two saturated fatty acids and one unsaturated fatty acid (SSU), TAGs esterified with one saturated fatty acid and two unsaturated fatty acids (SUU), and TAGs esterified with three unsaturated fatty acids (UUU). Figure 1B shows the contents of SSS, SSU, SUU, and UUU. The majority TAG structure was SUU, with a content of 52.88%, followed by SSU (32.05%), UUU (11.55%), and SSS (3.58%). SUU was composed of 38.93% UPU and 13.95% of other SSU.

### Identified MLCT molecules across the lactation stage and cities

The majority of MCFA exists in the form of MLCT in human milk. The identified MLCT molecules across the lactation stage and cities were presented in Supplementary Table 2.

A total of 60 kinds of MLCT molecules were identified, including 36 kinds of MLL-type TAGs and 24 kinds of MML-type TAGs. From the perspective of fatty acid composition, MLCTs were mainly composed of 12:0, 14:0, and long-chain fatty acids (16:0, 18:1, and 18:2). The most abundant MLCT molecule was O-P-La, followed by O-L-M, O-P-M, and O-O-M, which accounted for above 2% of total TAGs. The MLCTs with content over 1% in the present study were O-L-La, L-P-La, S-O-M, L-P-M, and O-M-La. Except for O-M-La, the other MLCTs with content over 1% were MLL types, indicating most MCFA existed in human milk in the form of MLL type.

As to the influence of lactation stages, 32 kinds were significantly influenced by lactation stages, such as O-P-M, O-O-M, and O-L-La. From colostrum to 1st month, the contents of O-P-M and O-O-M decreased significantly (*P* < 0.05), and from 1st month to 4th month, the contents remained stable at about 2%. The total content of MLCT in human milk in 1st month was the highest at 29.67%. A total of 37 kinds were significantly influenced by regional diversity, such as O-L-M, O-O-M, and O-L-La. The content of O-L-M in human milk in Jinan was significantly higher than that in Kunming and Xining human milk (*P* < 0.05). The content of O-L-La in human milk from Baotou, Jinan and Shenzhen (about 1.7%) was significantly higher than that in Kunming and Xining (*P* < 0.05). The total MLCT content in human milk from Baotou was the highest at 29.36%, which was significantly higher than that in Shenzhen and Jinan (*P* < 0.05). The interaction of lactation stage × region significantly influenced 21 kinds of MLCT, such as O-P-La, O-P-Ca, and L-L-La.

### Identified UPU molecules across the lactation stage and cities

The composition of UPU in human milk across lactation stages and regions was listed in Supplementary Table 3 in order of decreasing content. Human milk contained 15 kinds of UPU type TAGs. The typical UPU TAGs in human milk were O-P-L and O-P-O with content over 10%, followed by L-L-P and Et-L-P.

There were four kinds of UPU TAGs that were significantly (*P* < 0.05) influenced by the lactation stages, including L-L-P, O-P-Po, Ed-O-P, and Eo-L-P. The content of L-L-P showed a significant increasing trend with the prolonged lactation stages (*P* < 0.05). All UPU molecules were influenced by regional diversity (*P* < 0.05). O-L-P in human milk from Beijing, Shenzhen and Xining were significantly higher than that in the other three cities (*P* < 0.05). The content of O-O-P was significantly higher in Kunming and Xining than that in the other four cities (*P* < 0.05). The content of L-L-P in human milk from Kunming was the lowest. The interaction of the lactation stage and cities only significantly (*P* < 0.05) influenced three kinds of UPU TAGS, including Et-L-P, ARA-O-P, and Et-O-P.

## Discussion

### The sum content of MLCT and UPU in human milk was stable

MLCT and UPU in human milk provide better nutritional effects and should be prioritized as crucial focuses in neonatal nutrition research. However, little has been done on the influences of the lactation stage and regional diversity on MLCT and UPU.

**Figure 2:**
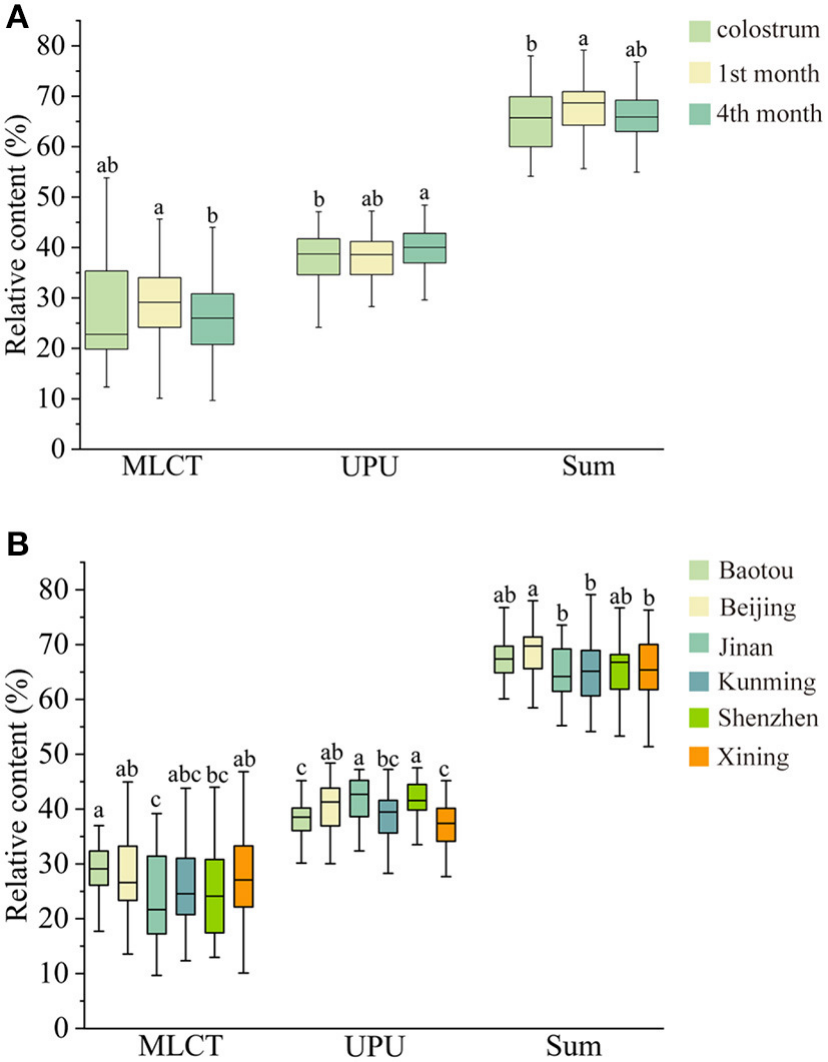
The content of MLCT, UPU, and the sum of MLCT and UPU in human milk at different lactation stages **(A)** and regions **(B)**. Different lowercase letters (a, b, c) indicate the significant differences (*P* < 0.05) across lactation stages or regions.

In this study, we explored the influence of the lactation stage and regional diversity on MLCT, UPU, and the sum of MLCT and UPU. As shown in Figure 2A, the average MLCT content was lowest in colostrum, and the MLCT in human milk in the 1st month was obviously higher than that in human milk in the 4th month (*P* < 0.05). The UPU content in human milk in the 4th month was the highest. The sum of MLCT and UPU accounted for 66.32 ± 5.27% (51.33–79.12%) of the total TAGs in human milk. As to the regional diversity, the human milk in Jinan contained the lowest MLCT content (23.55 ± 8.27%), and the sum of MLCT and UPU was highest in the milk of Beijing. Chen et al. (16) reported that the MLCT content in Zhengzhou and Wuhan was 35.86 and 29.26%, respectively. It was reported that the MLCT content was 42.29 and 53.21% in Beijing and Finland, respectively (17). Our data were slightly lower than these previous studies, which may be contributed to the regional diversity and analysis method. According to Figure 2, it seemed that the total content of MLCT, UPU and the sum content of MLCT and UPU in human milk were stable across lactation stages and cities. Moreover, we furtherly analyzed the relationship between TAG species and gender (Supplementary Figure 1). The OPLS-DA plot indicated that there was no significant difference in TAG species between boys and girls.

### MLCT was more associated with lactation stages

MLCT naturally existed in human milk, which had the potential to lower fat accumulation and facilitate fat absorption (4). Most MCFA existed in human milk in the form of MLL type. There was a significant gap between human milk and infant formula, as well as mammalian milk, which was abundant in MML types, such as P-M-Ca, P-M-La, and O-M-M (18, 19). The MLCT structure lipids have been prepared for better imitation of human milk lipids (20).

**Figure 3:**
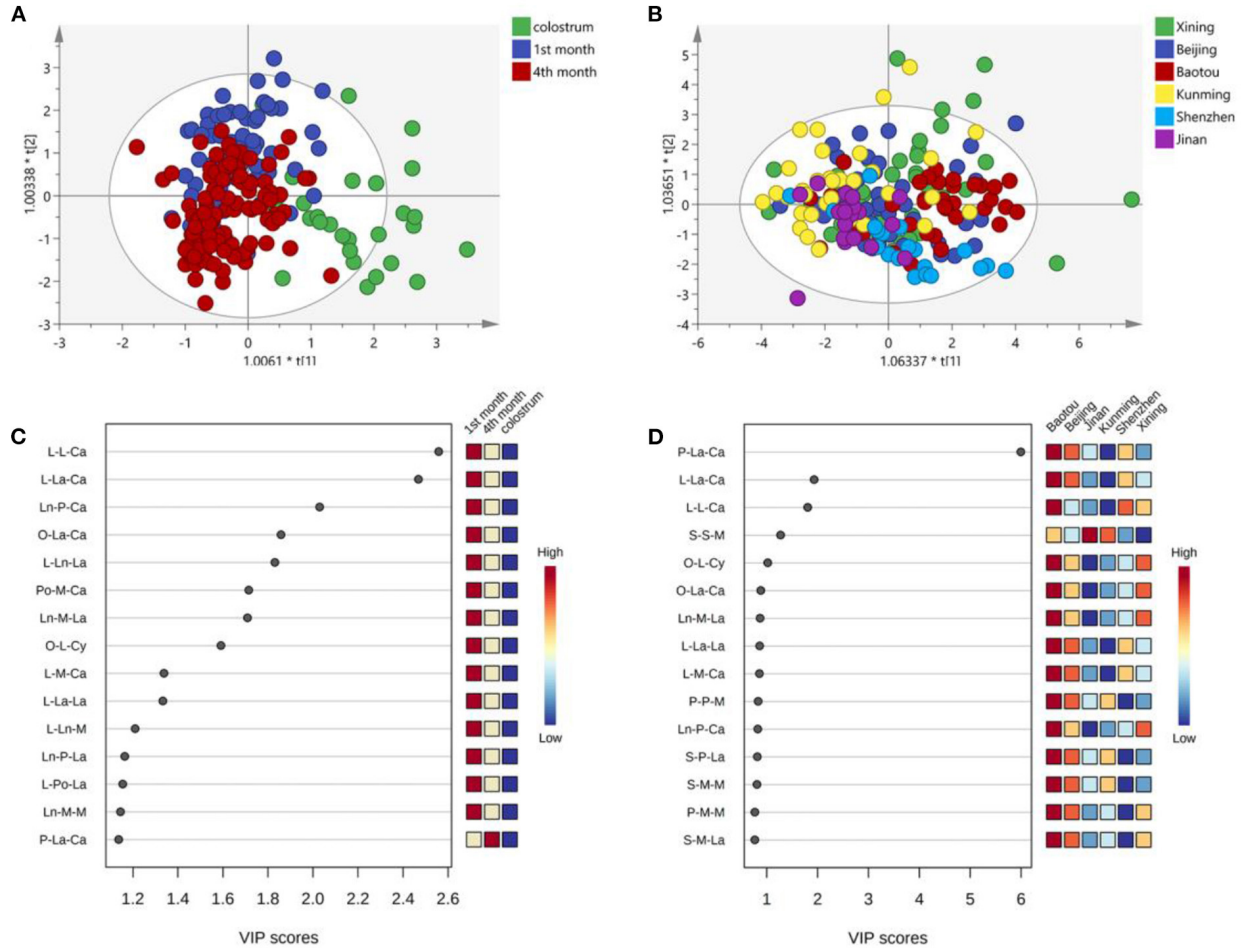
OPLS-DA analysis of MLCT types TAG across the lactation stage **(A)** and cities **(B)**; VIP scores plot for cluster analysis of MLCT molecules across the lactation stage **(C)** and cities **(D)**.

Figure 3 shows OPLS-DA analysis and VIP scores plot of MLCT molecules across the lactation stages and cities. From the perspective of the x-axis, the MLCT composition of human milk in the 1st month and the 4th month separated well-from that of colostrum (Figure 3A). As to the y-axis, the plots of the 1st month and the 4th month were overlapped, indicating a similar composition. As shown in Figure 3B, the plots of the six cities were scattered. Therefore, we could conclude that the MLCT composition was more influenced by the lactation stages than the regional diversity. According to the VIP scores plot, L-L-Ca, L-La-Ca, and Ln-P-Ca were the main contributing factors across the lactation stages, which were abundant in the human milk of the 1st month (Figure 3C). As to the regional diversity, the differences mainly existed in P-L-Ca, L-La-Ca, and L-L-Ca, which were higher in Beijing according to the VIP scores (Figure 3D).

### UPU was more associated with lactation stages

The *sn*-1 and *sn*-3 positions of UPU TAGs could be selectively hydrolyzed by pancreatic lipase in *vivo* to generate unsaturated fatty acid and monoglyceride containing 16:0, avoiding the combination with calcium (21). Therefore, UPU TAGs facilitate calcium absorption and soften the stool of infants. The UPU TAGs were mostly esterified with 16:0, 18:1 and 18:2. This phenomenon was consistent with our previous results (8, 22).

**Figure 4:**
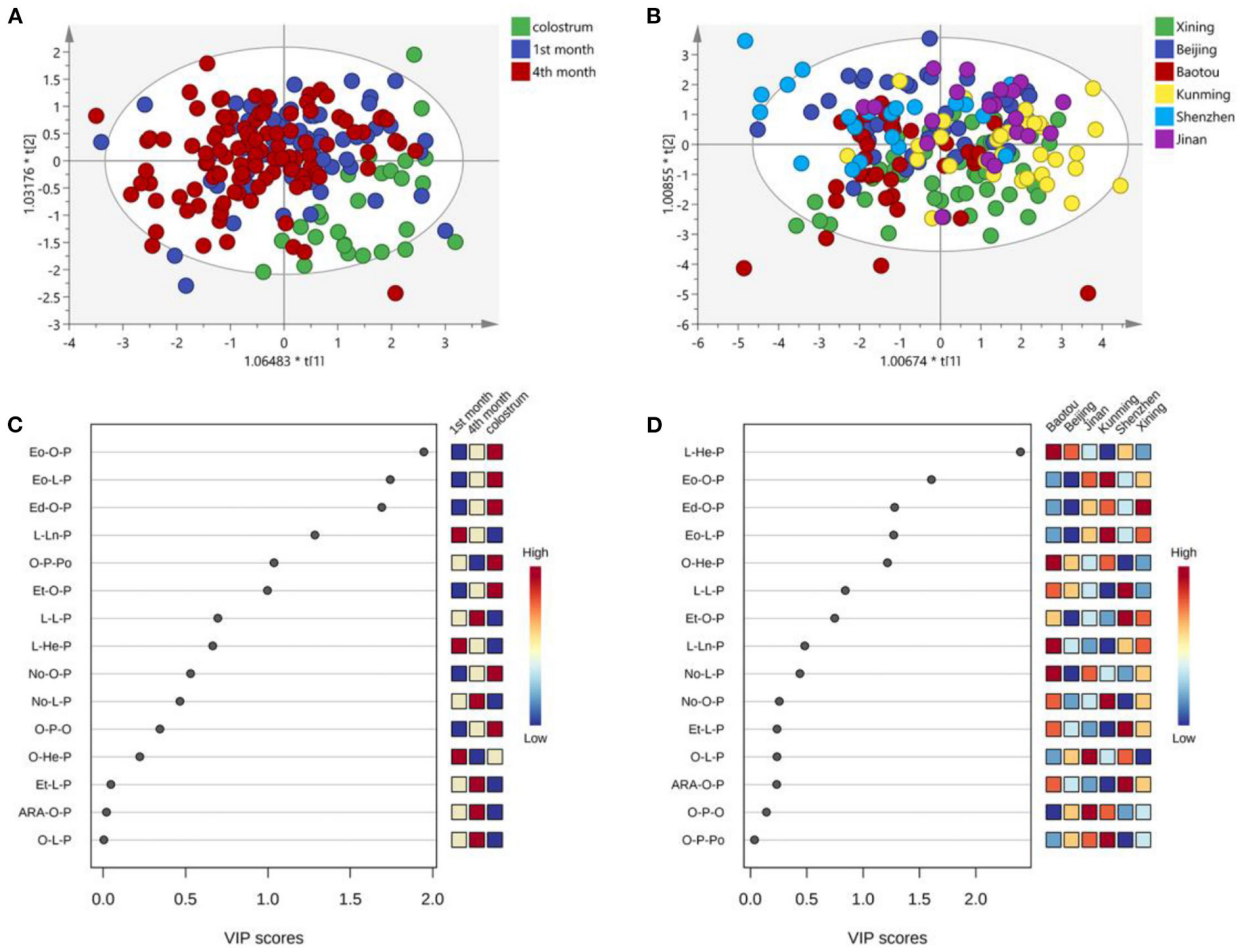
OPLS-DA analysis of UPU types TAG across the lactation stage **(A)** and cities **(B)**; VIP scores plot for cluster analysis of UPU molecules across the lactation stage **(C)** and cities **(D)**.

Figure 4 shows the OPLS-DA analysis and VIP scores plot of UPU molecules. The plots of the 1st and 4th months almost overlapped and separated well-with colostrum (Figure 4A). However, the UPU compositions of human milk in in the six cities were very close (Figure 4B). The lactation stages showed more obvious influences on the UPU composition than the regional diversity. The most varied UPU molecule across lactation stages was Eo-O-P, followed by Eo-L-P, and Ed-O-P, which were lowest in the milk in 1st month and highest in colostrum (Figure 4C). The differences across cities mainly existed in L-He-P, Eo-O-P, and Ed-O-P (Figure 4D). Both O-P-O and O-L-P contributed predominant variance of UPU compositions across lactation stages and cities.

### Chinese human milk was highly rich in O-P-L than western countries

**Figure 5:**
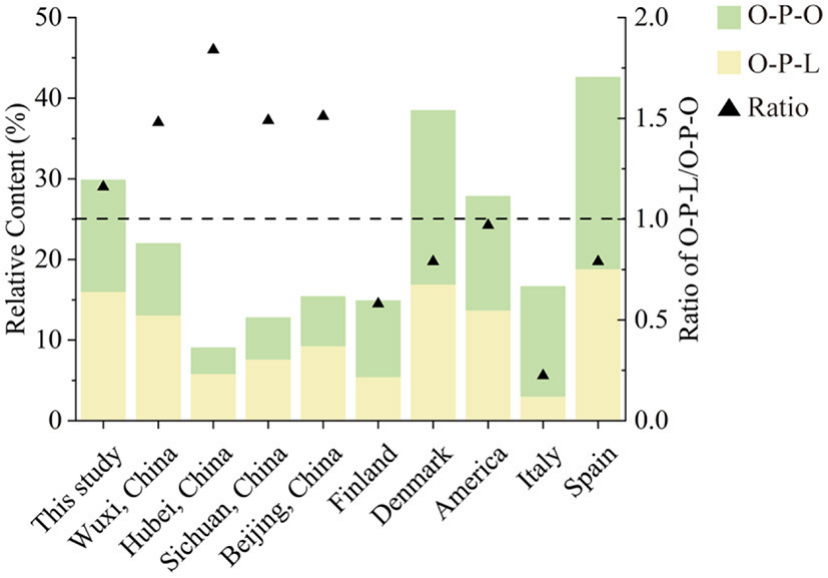
The contents of O-P-L, O-P-O, and the ratio of O-P-L to O-P-O in human milk from different countries.

O-P-L and O-P-O were the most abundant TAGs in human milk, which fits the results that most 16:0 was attached at the *sn*-2 position (23). This unique molecular structure of human milk TAGs promotes fat absorption in infants (3). We summarized the contents of O-P-L and O-P-O, and the ratio of O-P-L/O-P-O from different countries, including Wuxi, China (24), Hubei, Sichuan, and Beijing in China (25), Finland (17), Demark (26), America (27), Italy (28) and Spain (29). As shown in Figure 5, O-P-L (15–18%) was the most abundant TAG in China, followed by O-P-O (12–16%). However, O-P-O (9.43–23.73%) was higher than O-P-L (3.043–18.84%) in the other five countries. Therefore, the ratio of O-P-L to O-P-O ranged from 1.16 to 1.84 (>1) in Chinese human milk, while the ratio was 0.22–0.97 (<1) in western countries. This was in accordance with previous studies that reported the high concentration of linoleic acid in Chinese human milk (30).

The significant distinguishment reminded us to consider UPU TAGs in human milk, not a certain TAG, such as O-P-O or O-P-L. Dietary, genetic, sociodemographic, and environmental factors differences were considered to be the main reason for the variation of TAGs composition (17, 31). The higher O-P-L content in Chinese human milk was related to the higher consumption of linoleic acid in Chinese mothers. For example, olive oil with a content of oleic acid of more than 80% was widely used in cooking in western countries (32), while soybean oil and sunflower oil containing ~50–60% linoleic acid were consumed more in China (19, 25).

## Conclusions

In this paper, we investigated the influences of lactation stages and regional diversity on the characteristics of MLCT and UPU TAGs in human milk. The sum of MLCT and UPU was stable and accounted for ~70% of the total TAGs in human milk. MLCT was mainly composed of MLL-type TAGs. O-P-L and O-P-O were the predominant UPU TAGs. Both lactation stages and cities showed influences on MLCT and UPU molecules. Compared to regional diversity, lactation stages showed more influence on the composition of MLCT and UPU in human milk. Moreover, a summary of TAG studies indicated that Chinese human milk showed a higher ratio of O-P-L to O-P-O. These data provide a scientific basis for the two unique structure lipids, MLCT and UPU, in human milk.

## Data availability statement

The original contributions presented in the study are included in the article/Supplementary material, further inquiries can be directed to the corresponding author/s.

## Ethics statement

Written informed consent was obtained from the individual(s) for the publication of any potentially identifiable images or data included in this article.

## Author contributions

JY: writing - original draft and data analysis. ZY: validation and data analysis. LM: methodology and resources. LW: writing - original draft and formal analysis. ZL: conceptualization. XY: validation. QJ: data curation. JP: project administration. WW: conceptualization, methodology, validation, formal analysis, and writing - review. XW: project administration and supervision. All authors contributed to the article and approved the submitted version.

## Funding

This work was supported by the National Key R&D Program of China (2021YFD2100700) and the Major Science and Technology Special Project of Hohhot City Research on New Dairy-based Functional Ingredients and its Application in Infant Food.

## Conflict of interest

Authors LM and JP were employed by Inner Mongolia Mengniu Dairy (Group) Co., Ltd. ZY, ZL, and XY were employed by Inner Mongolia Mengniu Dairy (Group) Co., Ltd., and Yashili International Group Co., Ltd. The remaining authors declare that the research was conducted in the absence of any commercial or financial relationships that could be construed as a potential conflict of interest.

## Publisher's note

All claims expressed in this article are solely those of the authors and do not necessarily represent those of their affiliated organizations, or those of the publisher, the editors and the reviewers. Any product that may be evaluated in this article, or claim that may be made by its manufacturer, is not guaranteed or endorsed by the publisher.

## Abbreviations

TAG: triacylglycerol
MLCT: medium- and long-chain triacylglycerol
UPU: di-unsaturated fatty acyl-palmitoyl-glycerols
MCT: medium-chain triacylglycerol
HPLC: high-performance liquid chromatography
UPLC: ultra-performance liquid chromatography
Q-TOF-MS: coupled with quadrupole time-of-flight mass spectrometry.
