# Supplementary material for: Medium- and long-chain triacylglycerols and di-unsaturated fatty acyl-palmitoyl-glycerols in Chinese human milk: Association with region during the lactation
Source: Front Nutr. 2022 Oct 14;9:1040321. doi: 10.3389/fnut.2022.1040321 (PMC9614417; doi:10.3389/fnut.2022.1040321)

**Table S1.** Triacylglycerol compositions of human milk at different lactation periods and regions

| TAGs | Baotou | | Beijing | | | Jinan | Kunming | | | Shenzhen | Xining | | | Average |
| --- | --- | --- | --- | --- | --- | --- | --- | --- | --- | --- | --- | --- | --- | --- |
|  | 1^st^ month | 4^th^ month | Colostrum | 1^st^ month | 4^th^ month | 4^th^ month | Colostrum | 1^st^ month | 4^th^ month | 4^th^ month | Colostrum | 1^st^ month | 4^th^ month |  |
| M-La-Ca | 0.06±0.04 | 0.06±0.03 | 0.09±0.14 | 0.07±0.06 | 0.04±0.03 | 0.02±0.02 | 0.01±0.02 | 0.09±0.10 | 0.04±0.03 | 0.03±0.03 | 0.06±0.06 | 0.07±0.05 | 0.04±0.03 | 0.05±0.05 |
| La-La-La | 0.05±0.04 | 0.05±0.03 | 0.08±0.12 | 0.06±0.05 | 0.04±0.03 | 0.02±0.01 | 0.01±0.02 | 0.07±0.08 | 0.03±0.02 | 0.03±0.02 | 0.05±0.05 | 0.06±0.05 | 0.03±0.03 | 0.04±0.05 |
| P-La-Ca | 0.08±0.04 | 0.07±0.03 | 0.11±0.14 | 0.08±0.06 | 0.07±0.04 | 0.04±0.03 | 0.00±0.00 | 0.00±0.00 | 0.00±0.00 | 0.05±0.03 | 0.02±0.03 | 0.04±0.07 | 0.02±0.04 | 0.05±0.06 |
| M-M-Ca | 0.05±0.02 | 0.04±0.02 | 0.06±0.08 | 0.05±0.04 | 0.04±0.02 | 0.02±0.02 | 0.00±0.00 | 0.00±0.00 | 0.00±0.00 | 0.03±0.02 | 0.01±0.02 | 0.02±0.04 | 0.01±0.02 | 0.03±0.03 |
| M-La-La | 0.17±0.09 | 0.15±0.08 | 0.23±0.32 | 0.18±0.14 | 0.15±0.10 | 0.08±0.06 | 0.00±0.00 | 0.00±0.00 | 0.00±0.00 | 0.11±0.08 | 0.05±0.06 | 0.08±0.15 | 0.05±0.09 | 0.10±0.12 |
| L-La-Ca | 0.15±0.11 | 0.17±0.10 | 0.08±0.09 | 0.11±0.08 | 0.09±0.07 | 0.03±0.02 | 0.02±0.04 | 0.08±0.07 | 0.05±0.05 | 0.07±0.05 | 0.06±0.06 | 0.12±0.10 | 0.04±0.03 | 0.09±0.09 |
| O-La-Ca | 0.25±0.15 | 0.27±0.13 | 0.19±0.19 | 0.23±0.14 | 0.15±0.07 | 0.08±0.05 | 0.06±0.09 | 0.24±0.16 | 0.16±0.07 | 0.12±0.06 | 0.22±0.21 | 0.30±0.17 | 0.12±0.06 | 0.18±0.15 |
| Po-M-Ca | 0.03±0.02 | 0.03±0.01 | 0.02±0.02 | 0.03±0.02 | 0.02±0.01 | 0.01±0.01 | 0.01±0.01 | 0.03±0.02 | 0.02±0.01 | 0.01±0.01 | 0.02±0.02 | 0.03±0.02 | 0.01±0.01 | 0.02±0.02 |
| S-La-Ca | 0.07±0.03 | 0.06±0.03 | 0.10±0.14 | 0.08±0.05 | 0.07±0.04 | 0.05±0.04 | 0.03±0.03 | 0.10±0.09 | 0.06±0.03 | 0.05±0.04 | 0.08±0.06 | 0.07±0.05 | 0.06±0.04 | 0.07±0.05 |
| P-M-Ca | 0.15±0.06 | 0.13±0.06 | 0.21±0.27 | 0.16±0.11 | 0.14±0.08 | 0.09±0.08 | 0.05±0.06 | 0.20±0.19 | 0.12±0.05 | 0.11±0.08 | 0.16±0.13 | 0.14±0.09 | 0.13±0.08 | 0.13±0.11 |
| M-M-La | 0.29±0.11 | 0.25±0.11 | 0.41±0.53 | 0.31±0.21 | 0.27±0.16 | 0.18±0.15 | 0.10±0.11 | 0.38±0.37 | 0.23±0.1 | 0.20±0.15 | 0.31±0.25 | 0.26±0.18 | 0.25±0.15 | 0.26±0.21 |
| O-L-Co | 0.01±0.01 | 0.03±0.03 | 0.00±0.01 | 0.01±0.01 | 0.00±0.01 | 0.00±0.00 | 0.00±0.01 | 0.01±0.01 | 0.00±0.00 | 0.00±0.00 | 0.01±0.02 | 0.02±0.02 | 0.01±0.01 | 0.01±0.02 |
| L-M-Ca | 0.11±0.06 | 0.12±0.05 | 0.08±0.08 | 0.10±0.06 | 0.10±0.05 | 0.05±0.02 | 0.02±0.03 | 0.09±0.07 | 0.07±0.04 | 0.09±0.05 | 0.07±0.04 | 0.11±0.08 | 0.07±0.03 | 0.09±0.06 |
| L-La-La | 0.29±0.15 | 0.32±0.14 | 0.21±0.22 | 0.26±0.15 | 0.26±0.13 | 0.13±0.06 | 0.06±0.07 | 0.23±0.18 | 0.18±0.09 | 0.22±0.12 | 0.17±0.11 | 0.29±0.2 | 0.17±0.07 | 0.22±0.15 |
| O-M-Ca | 0.24±0.1 | 0.24±0.10 | 0.24±0.22 | 0.24±0.13 | 0.19±0.07 | 0.13±0.06 | 0.10±0.09 | 0.31±0.20 | 0.20±0.05 | 0.15±0.08 | 0.28±0.22 | 0.31±0.16 | 0.18±0.06 | 0.21±0.13 |
| O-La-La | 0.46±0.2 | 0.47±0.19 | 0.47±0.43 | 0.47±0.25 | 0.36±0.14 | 0.25±0.12 | 0.19±0.17 | 0.60±0.39 | 0.39±0.10 | 0.3±0.15 | 0.53±0.42 | 0.60±0.31 | 0.35±0.12 | 0.41±0.26 |
| Po-M-La | 0.11±0.05 | 0.12±0.05 | 0.12±0.11 | 0.12±0.06 | 0.09±0.04 | 0.06±0.03 | 0.05±0.04 | 0.15±0.10 | 0.10±0.03 | 0.07±0.04 | 0.13±0.11 | 0.15±0.08 | 0.09±0.03 | 0.10±0.06 |
| S-M-Ca | 0.09±0.03 | 0.08±0.03 | 0.11±0.12 | 0.09±0.05 | 0.08±0.04 | 0.07±0.06 | 0.04±0.04 | 0.11±0.08 | 0.08±0.03 | 0.07±0.05 | 0.09±0.07 | 0.07±0.04 | 0.08±0.04 | 0.08±0.05 |
| S-La-La | 0.1±0.03 | 0.08±0.03 | 0.12±0.13 | 0.10±0.06 | 0.09±0.05 | 0.07±0.06 | 0.04±0.04 | 0.11±0.08 | 0.08±0.03 | 0.07±0.05 | 0.09±0.07 | 0.08±0.04 | 0.08±0.05 | 0.08±0.06 |
| P-P-Ca | 0.11±0.03 | 0.09±0.04 | 0.14±0.15 | 0.12±0.07 | 0.10±0.05 | 0.09±0.07 | 0.05±0.05 | 0.13±0.10 | 0.09±0.04 | 0.08±0.06 | 0.11±0.08 | 0.09±0.05 | 0.10±0.05 | 0.10±0.07 |
| P-M-La | 0.43±0.12 | 0.35±0.14 | 0.52±0.56 | 0.44±0.25 | 0.39±0.20 | 0.32±0.26 | 0.18±0.19 | 0.50±0.37 | 0.35±0.13 | 0.31±0.22 | 0.41±0.31 | 0.34±0.18 | 0.37±0.20 | 0.37±0.25 |
| O-L-Cy | 0.02±0.02 | 0.03±0.02 | 0.01±0.01 | 0.01±0.01 | 0.01±0.01 | 0.00±0.00 | 0.00±0.01 | 0.01±0.01 | 0.01±0.00 | 0.01±0.01 | 0.02±0.02 | 0.02±0.01 | 0.01±0.01 | 0.01±0.02 |
| Ln-P-Ca | 0.05±0.04 | 0.07±0.05 | 0.02±0.03 | 0.03±0.03 | 0.02±0.02 | 0.01±0.01 | 0.01±0.02 | 0.02±0.03 | 0.01±0.01 | 0.01±0.02 | 0.04±0.04 | 0.05±0.03 | 0.03±0.03 | 0.03±0.04 |
| Ln-M-La | 0.03±0.02 | 0.04±0.03 | 0.01±0.01 | 0.02±0.02 | 0.01±0.01 | 0.00±0.00 | 0.00±0.01 | 0.01±0.01 | 0.01±0.01 | 0.01±0.01 | 0.02±0.02 | 0.03±0.02 | 0.01±0.01 | 0.02±0.02 |
| L-P-Ca | 0.68±0.23 | 0.67±0.23 | 0.50±0.39 | 0.66±0.24 | 0.67±0.26 | 0.42±0.16 | 0.19±0.13 | 0.58±0.35 | 0.46±0.18 | 0.58±0.26 | 0.44±0.21 | 0.62±0.31 | 0.45±0.14 | 0.55±0.27 |
| L-M-La | 0.36±0.12 | 0.36±0.12 | 0.26±0.21 | 0.35±0.13 | 0.35±0.14 | 0.22±0.08 | 0.10±0.07 | 0.31±0.18 | 0.25±0.09 | 0.31±0.14 | 0.23±0.11 | 0.33±0.17 | 0.24±0.07 | 0.29±0.14 |
| O-P-Ca | 0.76±0.2 | 0.71±0.21 | 0.73±0.52 | 0.81±0.31 | 0.67±0.26 | 0.54±0.23 | 0.36±0.29 | 1.04±0.50 | 0.74±0.17 | 0.56±0.26 | 0.85±0.48 | 0.88±0.36 | 0.64±0.21 | 0.70±0.34 |
| O-M-La | 1.44±0.39 | 1.34±0.40 | 1.38±0.99 | 1.54±0.58 | 1.26±0.49 | 1.03±0.43 | 0.68±0.55 | 1.98±0.94 | 1.40±0.32 | 1.06±0.49 | 1.61±0.91 | 1.67±0.69 | 1.20±0.39 | 1.33±0.64 |
| S-P-Ca | 0.11±0.03 | 0.09±0.03 | 0.11±0.09 | 0.11±0.05 | 0.10±0.04 | 0.09±0.07 | 0.05±0.04 | 0.11±0.05 | 0.09±0.03 | 0.08±0.05 | 0.09±0.05 | 0.07±0.03 | 0.09±0.04 | 0.09±0.05 |
| S-M-La | 0.36±0.09 | 0.30±0.10 | 0.38±0.31 | 0.35±0.16 | 0.32±0.13 | 0.30±0.23 | 0.18±0.15 | 0.35±0.16 | 0.30±0.10 | 0.26±0.17 | 0.30±0.18 | 0.24±0.11 | 0.29±0.15 | 0.30±0.16 |
| P-M-M | 0.51±0.13 | 0.42±0.14 | 0.53±0.43 | 0.50±0.23 | 0.45±0.19 | 0.43±0.32 | 0.25±0.21 | 0.50±0.23 | 0.42±0.14 | 0.37±0.24 | 0.42±0.26 | 0.34±0.16 | 0.42±0.21 | 0.43±0.23 |
| L-L-Ca | 0.15±0.08 | 0.20±0.10 | 0.03±0.03 | 0.09±0.06 | 0.07±0.08 | 0.03±0.02 | 0.01±0.04 | 0.03±0.06 | 0.02±0.03 | 0.11±0.07 | 0.05±0.06 | 0.15±0.10 | 0.06±0.05 | 0.09±0.09 |
| O-L-Ca | 0.30±0.11 | 0.34±0.11 | 0.16±0.10 | 0.25±0.10 | 0.20±0.07 | 0.12±0.04 | 0.08±0.08 | 0.16±0.11 | 0.15±0.06 | 0.22±0.08 | 0.21±0.11 | 0.32±0.12 | 0.2±0.06 | 0.22±0.12 |
| L-Po-La | 0.08±0.03 | 0.09±0.03 | 0.04±0.03 | 0.07±0.03 | 0.05±0.02 | 0.03±0.01 | 0.02±0.02 | 0.04±0.03 | 0.04±0.02 | 0.06±0.02 | 0.06±0.03 | 0.09±0.03 | 0.05±0.02 | 0.06±0.03 |
| Et-P-Ca | 0.03±0.01 | 0.03±0.01 | 0.02±0.01 | 0.03±0.01 | 0.02±0.01 | 0.01±0.00 | 0.01±0.01 | 0.02±0.01 | 0.02±0.01 | 0.02±0.01 | 0.02±0.01 | 0.03±0.01 | 0.02±0.01 | 0.02±0.01 |
| Ln-P-La | 0.13±0.05 | 0.15±0.05 | 0.07±0.04 | 0.11±0.04 | 0.09±0.03 | 0.05±0.02 | 0.04±0.03 | 0.07±0.05 | 0.06±0.03 | 0.09±0.03 | 0.09±0.05 | 0.14±0.05 | 0.09±0.03 | 0.10±0.05 |
| Ln-M-M | 0.05±0.02 | 0.05±0.02 | 0.03±0.02 | 0.04±0.02 | 0.03±0.01 | 0.02±0.01 | 0.01±0.01 | 0.03±0.02 | 0.02±0.01 | 0.04±0.01 | 0.03±0.02 | 0.05±0.02 | 0.03±0.01 | 0.04±0.02 |
| O-Po-La | 0.25±0.06 | 0.24±0.07 | 0.21±0.13 | 0.24±0.08 | 0.25±0.08 | 0.18±0.07 | 0.11±0.05 | 0.22±0.12 | 0.19±0.06 | 0.22±0.09 | 0.21±0.08 | 0.23±0.09 | 0.19±0.05 | 0.22±0.09 |
| L-P-La | 1.56±0.38 | 1.49±0.43 | 1.33±0.81 | 1.51±0.52 | 1.55±0.50 | 1.13±0.43 | 0.68±0.33 | 1.39±0.72 | 1.20±0.35 | 1.39±0.58 | 1.29±0.49 | 1.45±0.56 | 1.16±0.31 | 1.34±0.54 |
| L-M-M | 0.37±0.09 | 0.35±0.10 | 0.32±0.19 | 0.36±0.12 | 0.37±0.12 | 0.27±0.10 | 0.16±0.08 | 0.33±0.17 | 0.29±0.08 | 0.33±0.14 | 0.31±0.12 | 0.35±0.13 | 0.28±0.07 | 0.32±0.13 |
| O-P-La | 3.19±0.81 | 2.80±0.75 | 3.42±1.74 | 3.42±1.21 | 3.04±1.14 | 2.77±1.40 | 2.20±1.38 | 4.10±1.50 | 3.50±0.57 | 2.50±1.29 | 4.01±1.55 | 3.28±1.31 | 3.09±1.13 | 3.11±1.30 |
| O-M-M | 0.65±0.16 | 0.57±0.15 | 0.69±0.35 | 0.69±0.24 | 0.62±0.23 | 0.56±0.28 | 0.45±0.28 | 0.83±0.30 | 0.71±0.12 | 0.51±0.26 | 0.81±0.31 | 0.66±0.27 | 0.63±0.23 | 0.63±0.26 |
| S-P-La | 0.57±0.16 | 0.49±0.17 | 0.56±0.27 | 0.51±0.20 | 0.49±0.18 | 0.51±0.37 | 0.38±0.24 | 0.52±0.18 | 0.5±0.18 | 0.43±0.27 | 0.45±0.23 | 0.35±0.15 | 0.47±0.24 | 0.48±0.24 |
| S-M-M | 0.14±0.04 | 0.12±0.04 | 0.14±0.07 | 0.13±0.05 | 0.12±0.05 | 0.13±0.09 | 0.09±0.06 | 0.13±0.05 | 0.13±0.04 | 0.11±0.07 | 0.11±0.06 | 0.09±0.04 | 0.12±0.06 | 0.12±0.06 |
| P-P-M | 0.14±0.04 | 0.12±0.04 | 0.14±0.07 | 0.13±0.05 | 0.12±0.05 | 0.13±0.09 | 0.09±0.06 | 0.13±0.05 | 0.13±0.04 | 0.11±0.07 | 0.11±0.06 | 0.09±0.04 | 0.12±0.06 | 0.12±0.06 |
| L-Ln-La | 0.04±0.04 | 0.10±0.13 | 0.00±0.00 | 0.02±0.03 | 0.02±0.03 | 0.01±0.02 | 0.01±0.03 | 0.01±0.03 | 0.01±0.02 | 0.03±0.04 | 0.03±0.03 | 0.08±0.08 | 0.05±0.04 | 0.04±0.06 |
| L-L-La | 0.60±0.22 | 0.77±0.27 | 0.31±0.18 | 0.47±0.22 | 0.54±0.25 | 0.30±0.14 | 0.20±0.16 | 0.30±0.25 | 0.34±0.19 | 0.64±0.33 | 0.39±0.2 | 0.69±0.43 | 0.48±0.16 | 0.50±0.30 |
| O-L-La | 1.67±0.44 | 1.88±0.59 | 1.18±0.53 | 1.47±0.52 | 1.55±0.45 | 1.10±0.43 | 0.88±0.53 | 1.27±0.68 | 1.29±0.50 | 1.69±0.64 | 1.43±0.6 | 1.98±0.95 | 1.43±0.36 | 1.50±0.65 |
| L-Po-M | 0.22±0.06 | 0.25±0.08 | 0.16±0.07 | 0.20±0.07 | 0.21±0.06 | 0.15±0.06 | 0.12±0.07 | 0.17±0.09 | 0.17±0.07 | 0.23±0.09 | 0.19±0.08 | 0.26±0.13 | 0.19±0.05 | 0.20±0.09 |
| Po-Po-Po | 0.02±0.01 | 0.02±0.01 | 0.02±0.01 | 0.02±0.01 | 0.02±0.01 | 0.01±0.01 | 0.01±0.01 | 0.02±0.01 | 0.02±0.01 | 0.02±0.01 | 0.02±0.01 | 0.03±0.01 | 0.02±0.00 | 0.02±0.01 |
| Ed-M-M | 0.04±0.01 | 0.04±0.01 | 0.04±0.01 | 0.04±0.01 | 0.04±0.01 | 0.03±0.01 | 0.03±0.01 | 0.04±0.01 | 0.04±0.01 | 0.03±0.01 | 0.05±0.02 | 0.04±0.01 | 0.04±0.01 | 0.04±0.01 |
| O-O-La | 0.93±0.14 | 0.92±0.24 | 0.91±0.32 | 0.90±0.24 | 0.91±0.23 | 0.81±0.29 | 0.78±0.33 | 1.01±0.34 | 0.97±0.14 | 0.82±0.26 | 1.17±0.39 | 1.08±0.33 | 0.91±0.19 | 0.92±0.28 |
| O-Po-M | 0.10±0.02 | 0.10±0.03 | 0.10±0.04 | 0.10±0.03 | 0.10±0.03 | 0.09±0.03 | 0.09±0.04 | 0.11±0.04 | 0.11±0.02 | 0.09±0.03 | 0.13±0.04 | 0.12±0.04 | 0.10±0.02 | 0.10±0.03 |
| S-L-La | 0.62±0.09 | 0.62±0.16 | 0.61±0.21 | 0.60±0.16 | 0.61±0.15 | 0.54±0.19 | 0.52±0.22 | 0.67±0.23 | 0.65±0.10 | 0.55±0.18 | 0.78±0.26 | 0.72±0.22 | 0.61±0.12 | 0.62±0.19 |
| L-P-M | 1.13±0.17 | 1.12±0.29 | 1.10±0.39 | 1.09±0.30 | 1.10±0.28 | 0.98±0.35 | 0.95±0.40 | 1.22±0.41 | 1.18±0.17 | 1.00±0.32 | 1.41±0.47 | 1.31±0.40 | 1.11±0.22 | 1.12±0.34 |
| Eo-P-La | 0.06±0.02 | 0.05±0.01 | 0.08±0.02 | 0.06±0.02 | 0.05±0.02 | 0.06±0.03 | 0.07±0.03 | 0.07±0.02 | 0.07±0.02 | 0.05±0.03 | 0.09±0.03 | 0.05±0.02 | 0.06±0.02 | 0.06±0.02 |
| S-O-La | 0.55±0.16 | 0.47±0.12 | 0.68±0.20 | 0.52±0.16 | 0.47±0.19 | 0.52±0.25 | 0.64±0.27 | 0.63±0.21 | 0.62±0.16 | 0.42±0.22 | 0.80±0.27 | 0.47±0.17 | 0.51±0.17 | 0.54±0.22 |
| O-P-M | 2.21±0.64 | 1.9±0.47 | 2.71±0.81 | 2.08±0.65 | 1.90±0.74 | 2.08±1.00 | 2.56±1.09 | 2.52±0.85 | 2.49±0.63 | 1.69±0.89 | 3.19±1.06 | 1.90±0.69 | 2.03±0.68 | 2.15±0.86 |
| L-Ln-M | 0.06±0.03 | 0.11±0.10 | 0.02±0.03 | 0.04±0.04 | 0.04±0.03 | 0.01±0.03 | 0.02±0.04 | 0.03±0.04 | 0.03±0.03 | 0.04±0.05 | 0.06±0.05 | 0.07±0.07 | 0.06±0.05 | 0.05±0.06 |
| ARA-O-La | 0.01±0.01 | 0.01±0.01 | 0.02±0.02 | 0.01±0.01 | 0.00±0.00 | 0.00±0.00 | 0.01±0.01 | 0.01±0.01 | 0.00±0.00 | 0.00±0.01 | 0.01±0.01 | 0.01±0.01 | 0.00±0.00 | 0.01±0.01 |
| L-L-M | 0.71±0.18 | 0.83±0.26 | 0.51±0.16 | 0.57±0.19 | 0.68±0.24 | 0.47±0.16 | 0.45±0.15 | 0.42±0.25 | 0.49±0.21 | 0.74±0.32 | 0.65±0.19 | 0.68±0.29 | 0.61±0.14 | 0.62±0.25 |
| O-L-M | 2.33±0.41 | 2.37±0.56 | 2.22±0.40 | 2.16±0.36 | 2.25±0.57 | 1.95±0.49 | 2.15±0.39 | 1.89±0.65 | 2.01±0.6 | 2.27±0.64 | 2.56±0.55 | 2.11±0.63 | 1.91±0.30 | 2.16±0.55 |
| O-Po-Po | 0.48±0.08 | 0.49±0.12 | 0.46±0.08 | 0.45±0.08 | 0.47±0.12 | 0.40±0.10 | 0.45±0.08 | 0.39±0.13 | 0.42±0.12 | 0.47±0.13 | 0.53±0.11 | 0.44±0.13 | 0.40±0.06 | 0.45±0.11 |
| Eo-Po-M | 0.06±0.01 | 0.06±0.01 | 0.08±0.01 | 0.07±0.02 | 0.06±0.02 | 0.07±0.02 | 0.09±0.01 | 0.08±0.01 | 0.07±0.01 | 0.05±0.02 | 0.09±0.02 | 0.06±0.02 | 0.06±0.01 | 0.07±0.02 |
| O-O-M | 2.00±0.42 | 1.88±0.30 | 2.52±0.38 | 2.17±0.47 | 1.81±0.48 | 2.04±0.50 | 2.63±0.34 | 2.32±0.41 | 2.21±0.20 | 1.67±0.45 | 2.82±0.51 | 1.89±0.57 | 1.74±0.31 | 2.04±0.53 |
| O-P-Po | 0.93±0.21 | 0.89±0.16 | 1.20±0.20 | 1.01±0.25 | 0.81±0.24 | 0.97±0.27 | 1.26±0.16 | 1.11±0.20 | 1.06±0.10 | 0.77±0.22 | 1.38±0.27 | 0.92±0.3 | 0.83±0.15 | 0.96±0.27 |
| S-L-M | 0.74±0.15 | 0.63±0.16 | 0.81±0.13 | 0.80±0.16 | 0.76±0.15 | 0.67±0.20 | 0.82±0.11 | 0.72±0.13 | 0.69±0.06 | 0.62±0.15 | 0.79±0.17 | 0.54±0.15 | 0.55±0.13 | 0.68±0.18 |
| L-P-P | 1.82±0.36 | 1.54±0.39 | 1.98±0.32 | 1.95±0.39 | 1.85±0.38 | 1.64±0.49 | 2.01±0.26 | 1.77±0.32 | 1.69±0.15 | 1.53±0.38 | 1.93±0.41 | 1.31±0.36 | 1.36±0.32 | 1.68±0.43 |
| S-O-M | 1.15±0.32 | 0.98±0.29 | 1.51±0.28 | 1.32±0.41 | 1.09±0.38 | 1.21±0.37 | 1.50±0.34 | 1.33±0.42 | 1.28±0.32 | 0.88±0.37 | 1.24±0.40 | 0.85±0.33 | 0.96±0.31 | 1.13±0.4 |
| O-P-P | 4.06±1.13 | 3.46±1.02 | 5.35±0.99 | 4.67±1.47 | 3.86±1.35 | 4.26±1.31 | 5.30±1.20 | 4.70±1.48 | 4.51±1.12 | 3.13±1.32 | 4.38±1.42 | 3.01±1.16 | 3.41±1.09 | 3.98±1.42 |
| S-S-M | 0.04±0.01 | 0.04±0.02 | 0.04±0.01 | 0.04±0.01 | 0.04±0.01 | 0.04±0.02 | 0.04±0.01 | 0.04±0.02 | 0.04±0.01 | 0.03±0.01 | 0.02±0.01 | 0.02±0.01 | 0.03±0.01 | 0.03±0.01 |
| S-P-P | 0.50±0.18 | 0.48±0.22 | 0.48±0.09 | 0.47±0.12 | 0.45±0.17 | 0.49±0.2 | 0.5±0.15 | 0.48±0.19 | 0.48±0.18 | 0.39±0.16 | 0.31±0.18 | 0.27±0.13 | 0.32±0.12 | 0.43±0.19 |
| O-L-Pa | 0.06±0.02 | 0.06±0.02 | 0.04±0.01 | 0.05±0.02 | 0.04±0.02 | 0.04±0.02 | 0.03±0.01 | 0.03±0.01 | 0.04±0.02 | 0.04±0.02 | 0.04±0.01 | 0.03±0.02 | 0.03±0.03 | 0.04±0.02 |
| L-He-P | 0.04±0.01 | 0.04±0.01 | 0.02±0.00 | 0.03±0.01 | 0.03±0.01 | 0.02±0.01 | 0.02±0.01 | 0.02±0.01 | 0.02±0.01 | 0.02±0.01 | 0.03±0.01 | 0.02±0.01 | 0.02±0.02 | 0.03±0.01 |
| O-O-Pa | 0.03±0.01 | 0.03±0.02 | 0.02±0.01 | 0.03±0.01 | 0.02±0.01 | 0.03±0.01 | 0.03±0.01 | 0.03±0.01 | 0.03±0.01 | 0.02±0.01 | 0.03±0.01 | 0.02±0.01 | 0.02±0.01 | 0.03±0.01 |
| O-He-P | 0.09±0.03 | 0.09±0.06 | 0.07±0.01 | 0.08±0.03 | 0.06±0.03 | 0.07±0.04 | 0.07±0.02 | 0.07±0.02 | 0.08±0.02 | 0.05±0.03 | 0.08±0.03 | 0.06±0.04 | 0.06±0.04 | 0.07±0.04 |
| Ha-He-He | 0.02±0.01 | 0.02±0.01 | 0.02±0.00 | 0.02±0.01 | 0.01±0.01 | 0.02±0.01 | 0.02±0.00 | 0.02±0.00 | 0.02±0.01 | 0.01±0.01 | 0.02±0.01 | 0.01±0.01 | 0.01±0.01 | 0.02±0.01 |
| L-Ha-P | 0.07±0.03 | 0.07±0.05 | 0.05±0.01 | 0.06±0.03 | 0.05±0.02 | 0.06±0.03 | 0.06±0.01 | 0.05±0.01 | 0.06±0.02 | 0.04±0.02 | 0.07±0.03 | 0.05±0.03 | 0.05±0.03 | 0.06±0.03 |
| S-O-Pa | 0.03±0.02 | 0.04±0.04 | 0.02±0.01 | 0.03±0.01 | 0.02±0.01 | 0.03±0.02 | 0.03±0.01 | 0.03±0.01 | 0.03±0.01 | 0.02±0.01 | 0.03±0.02 | 0.02±0.02 | 0.03±0.02 | 0.03±0.02 |
| O-Ha-P | 0.13±0.08 | 0.17±0.18 | 0.09±0.02 | 0.11±0.06 | 0.08±0.04 | 0.10±0.07 | 0.10±0.03 | 0.10±0.04 | 0.12±0.06 | 0.06±0.05 | 0.11±0.08 | 0.09±0.09 | 0.1±0.08 | 0.11±0.09 |
| L-Ln-Po | 0.01±0.01 | 0.03±0.05 | 0.00±0.00 | 0.00±0.01 | 0.00±0.00 | 0.00±0.00 | 0.00±0.00 | 0.00±0.00 | 0.00±0.00 | 0.00±0.01 | 0.00±0.01 | 0.01±0.01 | 0.01±0.02 | 0.01±0.02 |
| L-L-Po | 0.18±0.06 | 0.25±0.16 | 0.11±0.02 | 0.17±0.06 | 0.15±0.05 | 0.11±0.05 | 0.11±0.04 | 0.09±0.07 | 0.11±0.05 | 0.15±0.06 | 0.14±0.04 | 0.19±0.05 | 0.18±0.09 | 0.16±0.09 |
| L-Ln-P | 0.55±0.17 | 0.77±0.48 | 0.33±0.07 | 0.51±0.18 | 0.46±0.16 | 0.34±0.16 | 0.32±0.11 | 0.29±0.20 | 0.32±0.14 | 0.47±0.19 | 0.43±0.13 | 0.56±0.16 | 0.54±0.27 | 0.48±0.26 |
| O-L-Po | 0.39±0.08 | 0.44±0.10 | 0.28±0.04 | 0.40±0.12 | 0.45±0.11 | 0.35±0.10 | 0.27±0.07 | 0.26±0.12 | 0.29±0.09 | 0.48±0.14 | 0.27±0.08 | 0.32±0.08 | 0.34±0.10 | 0.37±0.12 |
| L-L-P | 5.64±1.18 | 6.36±1.49 | 3.97±0.61 | 5.72±1.68 | 6.46±1.52 | 4.98±1.38 | 3.88±0.97 | 3.70±1.66 | 4.12±1.34 | 6.85±2.01 | 3.93±1.11 | 4.64±1.17 | 4.88±1.42 | 5.25±1.76 |
| O-L-P | 16.1±1.49 | 14.63±2.16 | 16.16±2.8 | 16.92±2.09 | 17.91±2.00 | 17.49±1.98 | 15.94±2.17 | 15.3±2.38 | 15.63±1.76 | 17.25±1.82 | 14.45±2.9 | 14.55±1.96 | 15.14±1.32 | 16.04±2.32 |
| O-P-O | 12.90±1.40 | 12.01±1.95 | 14.41±3.11 | 14.00±2.20 | 13.80±2.05 | 15.77±2.27 | 15.5±1.94 | 15.4±3.33 | 15.49±1.57 | 12.89±2.2 | 13.61±2.68 | 13.06±2.65 | 14.18±2.00 | 13.90±2.52 |
| S-L-P | 3.89±0.42 | 3.62±0.59 | 4.34±0.94 | 4.22±0.66 | 4.16±0.62 | 4.75±0.69 | 4.67±0.59 | 4.64±1.00 | 4.67±0.47 | 3.89±0.66 | 4.10±0.81 | 3.94±0.80 | 4.27±0.60 | 4.19±0.76 |
| S-O-P | 4.80±1.92 | 5.05±2.68 | 4.40±1.18 | 4.53±1.18 | 4.70±1.72 | 6.10±2.10 | 5.24±1.27 | 6.60±2.98 | 6.81±2.50 | 4.07±1.67 | 3.46±1.46 | 3.74±1.78 | 4.86±1.29 | 4.90±2.12 |
| S-S-P | 0.27±0.13 | 0.32±0.26 | 0.18±0.04 | 0.21±0.08 | 0.24±0.10 | 0.29±0.15 | 0.22±0.07 | 0.30±0.16 | 0.31±0.15 | 0.23±0.09 | 0.11±0.07 | 0.16±0.1 | 0.19±0.08 | 0.24±0.15 |
| No-L-P | 0.02±0.01 | 0.03±0.02 | 0.01±0.01 | 0.02±0.01 | 0.02±0.01 | 0.02±0.01 | 0.02±0.01 | 0.01±0.00 | 0.02±0.00 | 0.02±0.01 | 0.02±0.01 | 0.02±0.01 | 0.02±0.01 | 0.02±0.01 |
| O-L-Ha | 0.11±0.05 | 0.13±0.07 | 0.07±0.03 | 0.09±0.05 | 0.08±0.02 | 0.09±0.05 | 0.09±0.04 | 0.07±0.02 | 0.09±0.02 | 0.08±0.03 | 0.1±0.03 | 0.09±0.06 | 0.09±0.06 | 0.09±0.05 |
| No-O-P | 0.02±0.01 | 0.04±0.04 | 0.02±0.01 | 0.02±0.01 | 0.01±0.01 | 0.02±0.02 | 0.03±0.01 | 0.02±0.01 | 0.03±0.01 | 0.01±0.01 | 0.03±0.01 | 0.02±0.02 | 0.03±0.02 | 0.02±0.02 |
| S-O-He | 0.05±0.03 | 0.09±0.10 | 0.04±0.02 | 0.05±0.03 | 0.04±0.02 | 0.06±0.04 | 0.06±0.02 | 0.06±0.02 | 0.07±0.02 | 0.03±0.02 | 0.07±0.03 | 0.06±0.05 | 0.06±0.05 | 0.06±0.05 |
| O-O-Ha | 0.02±0.01 | 0.04±0.04 | 0.02±0.01 | 0.02±0.01 | 0.01±0.01 | 0.02±0.02 | 0.03±0.01 | 0.02±0.01 | 0.03±0.01 | 0.01±0.01 | 0.03±0.01 | 0.02±0.02 | 0.03±0.02 | 0.02±0.02 |
| L-L-Ln | 0.12±0.09 | 0.33±0.42 | 0.01±0.02 | 0.08±0.08 | 0.08±0.13 | 0.05±0.07 | 0.03±0.06 | 0.03±0.06 | 0.02±0.04 | 0.13±0.12 | 0.06±0.04 | 0.24±0.22 | 0.21±0.18 | 0.13±0.2 |
| L-L-L | 1.15±0.45 | 1.67±0.70 | 0.52±0.13 | 0.87±0.43 | 1.05±0.87 | 0.71±0.44 | 0.65±0.29 | 0.38±0.42 | 0.50±0.31 | 1.67±0.93 | 0.68±0.21 | 1.54±1.14 | 1.36±0.76 | 1.09±0.8 |
| Et-L-P | 1.16±0.39 | 1.45±0.40 | 0.73±0.18 | 0.95±0.36 | 1.10±0.70 | 0.91±0.46 | 0.98±0.35 | 0.52±0.39 | 0.63±0.29 | 1.81±0.83 | 0.83±0.23 | 1.40±0.80 | 1.37±0.71 | 1.15±0.65 |
| O-L-L | 2.44±0.82 | 3.03±0.84 | 1.53±0.37 | 1.99±0.76 | 2.30±1.47 | 1.91±0.96 | 2.05±0.73 | 1.08±0.82 | 1.31±0.61 | 3.78±1.73 | 1.75±0.49 | 2.92±1.67 | 2.86±1.49 | 2.4±1.37 |
| ARA-O-P | 0.28±0.09 | 0.35±0.10 | 0.18±0.04 | 0.23±0.09 | 0.26±0.17 | 0.22±0.11 | 0.24±0.08 | 0.12±0.09 | 0.15±0.07 | 0.43±0.20 | 0.20±0.06 | 0.34±0.19 | 0.33±0.17 | 0.28±0.16 |
| O-O-L | 4.38±0.96 | 4.82±1.19 | 4.27±1.27 | 3.97±1.02 | 4.34±1.75 | 4.50±1.73 | 5.87±1.32 | 3.12±1.20 | 3.83±1.21 | 5.81±1.93 | 4.62±1.24 | 5.58±2.29 | 5.73±2.01 | 4.78±1.77 |
| Et-O-P | 0.14±0.03 | 0.15±0.04 | 0.13±0.04 | 0.13±0.03 | 0.14±0.06 | 0.14±0.05 | 0.19±0.04 | 0.1±0.04 | 0.12±0.04 | 0.18±0.06 | 0.15±0.04 | 0.18±0.07 | 0.18±0.06 | 0.15±0.06 |
| S-L-L | 1.02±0.22 | 1.12±0.27 | 0.99±0.3 | 0.92±0.24 | 1.01±0.40 | 1.04±0.40 | 1.36±0.31 | 0.72±0.28 | 0.89±0.28 | 1.35±0.45 | 1.07±0.29 | 1.29±0.53 | 1.33±0.46 | 1.11±0.41 |
| Ed-O-P | 0.37±0.08 | 0.43±0.10 | 0.48±0.19 | 0.35±0.07 | 0.39±0.10 | 0.52±0.21 | 0.71±0.17 | 0.45±0.14 | 0.53±0.17 | 0.45±0.15 | 0.53±0.15 | 0.57±0.24 | 0.6±0.18 | 0.48±0.18 |
| O-O-O | 1.62±0.33 | 1.84±0.45 | 2.09±0.80 | 1.53±0.32 | 1.68±0.45 | 2.27±0.91 | 3.06±0.73 | 1.94±0.61 | 2.31±0.73 | 1.97±0.64 | 2.31±0.64 | 2.46±1.05 | 2.59±0.77 | 2.1±0.79 |
| Eo-L-P | 0.06±0.01 | 0.07±0.02 | 0.08±0.03 | 0.06±0.01 | 0.06±0.02 | 0.08±0.03 | 0.11±0.03 | 0.07±0.02 | 0.08±0.03 | 0.07±0.02 | 0.08±0.02 | 0.09±0.04 | 0.09±0.03 | 0.08±0.03 |
| S-O-L | 1.69±0.35 | 1.93±0.47 | 2.19±0.84 | 1.60±0.34 | 1.75±0.47 | 2.38±0.95 | 3.20±0.76 | 2.03±0.64 | 2.41±0.76 | 2.06±0.67 | 2.42±0.67 | 2.57±1.10 | 2.71±0.80 | 2.19±0.83 |
| Eo-S-P | 0.12±0.04 | 0.15±0.07 | 0.15±0.05 | 0.11±0.03 | 0.13±0.03 | 0.19±0.06 | 0.22±0.05 | 0.19±0.07 | 0.21±0.07 | 0.14±0.05 | 0.17±0.04 | 0.19±0.09 | 0.21±0.09 | 0.16±0.07 |
| S-O-O | 0.97±0.29 | 1.20±0.59 | 1.19±0.43 | 0.87±0.20 | 0.99±0.24 | 1.50±0.50 | 1.76±0.42 | 1.49±0.56 | 1.67±0.53 | 1.10±0.39 | 1.37±0.32 | 1.50±0.69 | 1.67±0.75 | 1.3±0.57 |
| E-L-P | 0.02±0.01 | 0.03±0.01 | 0.03±0.01 | 0.02±0.00 | 0.02±0.01 | 0.04±0.01 | 0.04±0.01 | 0.04±0.01 | 0.04±0.01 | 0.03±0.01 | 0.03±0.01 | 0.04±0.02 | 0.04±0.02 | 0.03±0.01 |
| Eo-O-P | 0.01±0.00 | 0.02±0.01 | 0.02±0.01 | 0.01±0.00 | 0.01±0.00 | 0.02±0.01 | 0.03±0.01 | 0.02±0.01 | 0.02±0.01 | 0.02±0.01 | 0.02±0.00 | 0.02±0.01 | 0.02±0.01 | 0.02±0.01 |
| Ed-S-P | 0.02±0.00 | 0.02±0.01 | 0.02±0.01 | 0.01±0.00 | 0.02±0.00 | 0.02±0.01 | 0.03±0.01 | 0.02±0.01 | 0.03±0.01 | 0.02±0.01 | 0.02±0.01 | 0.02±0.01 | 0.03±0.01 | 0.02±0.01 |
| S-S-L | 0.15±0.05 | 0.19±0.09 | 0.19±0.07 | 0.13±0.03 | 0.15±0.04 | 0.23±0.08 | 0.27±0.07 | 0.23±0.09 | 0.26±0.08 | 0.17±0.06 | 0.21±0.05 | 0.23±0.11 | 0.26±0.12 | 0.20±0.09 |
| E-O-P | 0.08±0.03 | 0.09±0.07 | 0.07±0.03 | 0.06±0.02 | 0.08±0.02 | 0.12±0.04 | 0.10±0.03 | 0.1±0.05 | 0.12±0.05 | 0.09±0.04 | 0.07±0.02 | 0.10±0.06 | 0.11±0.04 | 0.09±0.05 |
| S-S-O | 0.17±0.08 | 0.20±0.15 | 0.16±0.06 | 0.13±0.04 | 0.17±0.05 | 0.26±0.10 | 0.22±0.06 | 0.21±0.10 | 0.25±0.11 | 0.20±0.08 | 0.16±0.05 | 0.21±0.13 | 0.24±0.10 | 0.20±0.10 |
| E-O-L | 0.04±0.02 | 0.05±0.04 | 0.1±0.06 | 0.03±0.02 | 0.03±0.02 | 0.06±0.04 | 0.16±0.04 | 0.06±0.03 | 0.06±0.03 | 0.06±0.03 | 0.15±0.03 | 0.16±0.13 | 0.14±0.15 | 0.08±0.08 |
| Eo-S-L | 0.06±0.04 | 0.09±0.06 | 0.17±0.09 | 0.05±0.03 | 0.05±0.03 | 0.1±0.06 | 0.26±0.07 | 0.09±0.05 | 0.1±0.05 | 0.09±0.05 | 0.24±0.05 | 0.26±0.21 | 0.22±0.25 | 0.13±0.14 |
| Eo-O-O | 0.03±0.02 | 0.04±0.03 | 0.08±0.04 | 0.03±0.01 | 0.02±0.01 | 0.05±0.03 | 0.12±0.03 | 0.04±0.02 | 0.05±0.02 | 0.04±0.02 | 0.11±0.02 | 0.12±0.10 | 0.10±0.12 | 0.06±0.06 |
| Do-L-P | 0.03±0.03 | 0.05±0.05 | 0.07±0.04 | 0.02±0.02 | 0.02±0.02 | 0.07±0.04 | 0.12±0.03 | 0.06±0.05 | 0.07±0.06 | 0.05±0.04 | 0.15±0.05 | 0.23±0.26 | 0.2±0.35 | 0.09±0.16 |
| E-S-L | 0.05±0.04 | 0.07±0.07 | 0.1±0.06 | 0.04±0.03 | 0.03±0.03 | 0.09±0.06 | 0.18±0.04 | 0.09±0.07 | 0.10±0.08 | 0.08±0.06 | 0.22±0.07 | 0.32±0.38 | 0.29±0.50 | 0.13±0.22 |
| Do-O-P | 0.01±0.02 | 0.01±0.02 | 0.01±0.02 | 0.01±0.01 | 0.02±0.02 | 0.05±0.05 | 0.03±0.02 | 0.01±0.01 | 0.01±0.02 | 0.05±0.05 | 0.03±0.03 | 0.04±0.05 | 0.04±0.06 | 0.03±0.04 |
| E-S-O | 0.01±0.01 | 0.01±0.01 | 0.01±0.01 | 0.00±0.00 | 0.01±0.01 | 0.03±0.03 | 0.02±0.01 | 0.01±0.01 | 0.01±0.01 | 0.03±0.03 | 0.02±0.02 | 0.02±0.03 | 0.02±0.03 | 0.01±0.02 |
| SLCT | 0.01±0.01 | 0.03±0.03 | 0.00±0.01 | 0.01±0.01 | 0.00±0.01 | 0.00±0.00 | 0.00±0.01 | 0.01±0.01 | 0.00±0.00 | 0.00±0.00 | 0.01±0.02 | 0.02±0.02 | 0.01±0.01 | 0.01±0.02 |
| MCT | 0.63±0.29 | 0.54±0.27 | 0.87±1.19 | 0.67±0.48 | 0.53±0.33 | 0.32±0.26 | 0.12±0.14 | 0.55±0.55 | 0.31±0.14 | 0.40±0.28 | 0.48±0.33 | 0.49±0.45 | 0.39±0.28 | 0.48±0.43 |
| MLCT | 29.90±5.04 | 28.82±5.99 | 29.41±12.17 | 29.28±7.66 | 27.29±6.97 | 23.55±8.27 | 21.59±7.74 | 30.65±10.19 | 27.34±4.1 | 24.72±8.65 | 32.13±9.19 | 29.20±9.21 | 25.20±5.89 | 27.39±8.21 |
| LCT | 69.46±5.24 | 70.61±6.24 | 69.72±13.23 | 70.05±8.09 | 72.18±7.24 | 76.13±8.5 | 78.29±7.88 | 68.8±10.7 | 72.35±4.22 | 74.87±8.92 | 67.38±9.50 | 70.29±9.54 | 74.40±6.14 | 72.12±8.55 |
| MLL | 23.38±3.63 | 22.73±4.26 | 23.04±7.21 | 22.77±5.04 | 21.63±5.05 | 19.18±6.15 | 18.79±5.68 | 23.40±6.51 | 21.95±3.02 | 20.01±6.36 | 25.9±6.11 | 22.76±6.54 | 20.17±4.11 | 21.78±5.68 |
| MML | 6.52±1.68 | 6.09±1.85 | 6.37±5.18 | 6.51±2.75 | 5.66±2.10 | 4.37±2.19 | 2.79±2.12 | 7.25±3.84 | 5.39±1.18 | 4.72±2.36 | 6.23±3.27 | 6.44±2.83 | 5.03±1.82 | 5.61±2.72 |
| UPU | 38.32±2.76 | 37.30±3.59 | 37.81±6.75 | 40.03±4.81 | 41.51±3.96 | 41.59±4.47 | 39.30±4.49 | 37.20±5.46 | 38.31±2.84 | 41.3±4.51 | 35.78±6.02 | 36.44±4.15 | 38.29±2.95 | 38.93±4.67 |
| MLCT+UPU | 68.22±3.83 | 66.13±4.78 | 67.21±6.09 | 69.31±3.55 | 68.8±4.17 | 65.14±4.87 | 60.88±4.31 | 67.85±5.62 | 65.65±4.34 | 66.02±5.22 | 67.91±4.79 | 65.65±6.49 | 63.49±4.46 | 66.32±5.27 |

NS, *P*>0.05; *, *P*<0.05; **, *P*<0.01; ***, *P*<0.001. Co, 6:0; Cy, 8:0; Ca, 10:0; La, 12:0; M, 14:0; Pa, 15:0; P, 16:0; Po, 16:1; Ha, 17:0; He, 17:1; O, 18:1; S, 18:0; L, 18:2; Ln, 18:3; No, 19:1; E, 20:0; Eo, 20:1; Ed, 20:2; Et, 20:3; ARA, 20:4; Do, 22:0.

**Table S2.** Composition of MLCT (wt %) in human milk at different lactation periods and regions

| TAGs | Lactational periods | Baotou | Beijing | Jinan | Kunming | Shenzhen | Xining | P1 (lactations) | P2 (cities) | P1*P2 |
| --- | --- | --- | --- | --- | --- | --- | --- | --- | --- | --- |
| MLL type |  |  |  |  |  |  |  |  |  |  |
| O-P-La | Colostrum | - | 3.42±1.74 | - | 2.20±1.38 | - | 4.01±1.55 | NS | NS | * |
|  | 1^st^ month | 3.19±0.81 | 3.42±1.21 | - | 4.10±1.50 | - | 3.28±1.31 |  |  |  |
|  | 4^th^ month | 2.80±0.75 | 3.04±1.14 | 2.77±1.40 | 3.50±0.57 | 2.50±1.29 | 3.09±1.13 |  |  |  |
| O-L-M | Colostrum | - | 2.22±0.40 | - | 2.15±0.39 | - | 2.56±0.55 | NS | * | NS |
|  | 1^st^ month | 2.33±0.41 | 2.16±0.36 | - | 1.89±0.65 | - | 2.11±0.63 |  |  |  |
|  | 4^th^ month | 2.37±0.56 | 2.25±0.57 | 1.95±0.49 | 2.01±0.60 | 2.27±0.64 | 1.91±0.30 |  |  |  |
| O-P-M | Colostrum | - | 2.71±0.81 | - | 2.56±1.09 | - | 3.19±1.06 | *** | NS | NS |
|  | 1^st^ month | 2.21±0.64 | 2.08±0.65 | - | 2.52±0.85 | - | 1.90±0.69 |  |  |  |
|  | 4^th^ month | 1.90±0.47 | 1.90±0.74 | 2.08±1.00 | 2.49±0.63 | 1.69±0.89 | 2.03±0.68 |  |  |  |
| O-O-M | Colostrum | - | 2.52±0.38 | - | 2.63±0.34 | - | 2.82±0.51 | *** | * | NS |
|  | 1^st^ month | 2.00±0.42 | 2.17±0.47 | - | 2.32±0.41 | - | 1.89±0.57 |  |  |  |
|  | 4^th^ month | 1.88±0.30 | 1.81±0.48 | 2.04±0.50 | 2.21±0.20 | 1.67±0.45 | 1.74±0.31 |  |  |  |
| O-L-La | Colostrum | - | 1.18±0.53 | - | 0.88±0.53 | - | 1.43±0.60 | * | *** | NS |
|  | 1^st^ month | 1.67±0.44 | 1.47±0.52 | - | 1.27±0.68 | - | 1.98±0.95 |  |  |  |
|  | 4^th^ month | 1.88±0.59 | 1.55±0.45 | 1.10±0.43 | 1.29±0.50 | 1.69±0.64 | 1.43±0.36 |  |  |  |
| L-P-La | Colostrum | - | 1.33±0.81 | - | 0.68±0.33 | - | 1.29±0.49 | * | * | NS |
|  | 1^st^ month | 1.56±0.38 | 1.51±0.52 | - | 1.39±0.72 | - | 1.45±0.56 |  |  |  |
|  | 4^th^ month | 1.49±0.43 | 1.55±0.50 | 1.13±0.43 | 1.20±0.35 | 1.39±0.58 | 1.16±0.31 |  |  |  |
| S-O-M | Colostrum | - | 1.51±0.28 | - | 1.50±0.34 | - | 1.24±0.40 | *** | *** | NS |
|  | 1^st^ month | 1.15±0.32 | 1.32±0.41 | - | 1.33±0.42 | - | 0.85±0.33 |  |  |  |
|  | 4^th^ month | 0.98±0.29 | 1.09±0.38 | 1.21±0.37 | 1.28±0.32 | 0.88±0.37 | 0.96±0.31 |  |  |  |
| L-P-M | Colostrum | - | 1.10±0.39 | - | 0.95±0.40 | - | 1.41±0.47 | NS | * | NS |
|  | 1^st^ month | 1.13±0.17 | 1.09±0.30 | - | 1.22±0.41 | - | 1.31±0.40 |  |  |  |
|  | 4^th^ month | 1.12±0.29 | 1.10±0.28 | 0.98±0.35 | 1.18±0.17 | 1.00±0.32 | 1.11±0.22 |  |  |  |
| O-O-La | Colostrum | - | 0.91±0.32 | - | 0.78±0.33 | - | 1.17±0.39 | NS | * | NS |
|  | 1^st^ month | 0.93±0.14 | 0.90±0.24 | - | 1.01±0.34 | - | 1.08±0.33 |  |  |  |
|  | 4^th^ month | 0.92±0.24 | 0.91±0.23 | 0.81±0.29 | 0.97±0.14 | 0.82±0.26 | 0.91±0.19 |  |  |  |
| O-P-Ca | Colostrum | - | 0.73±0.52 | - | 0.36±0.29 | - | 0.85±0.48 | *** | NS | ** |
|  | 1^st^ month | 0.76±0.20 | 0.81±0.31 | - | 1.04±0.50 | - | 0.88±0.36 |  |  |  |
|  | 4^th^ month | 0.71±0.21 | 0.67±0.26 | 0.54±0.23 | 0.74±0.17 | 0.56±0.26 | 0.64±0.21 |  |  |  |
| S-L-M | Colostrum | - | 0.81±0.13 | - | 0.82±0.11 | - | 0.79±0.17 | *** | *** | NS |
|  | 1^st^ month | 0.74±0.15 | 0.80±0.16 | - | 0.72±0.13 | - | 0.54±0.15 |  |  |  |
|  | 4^th^ month | 0.63±0.16 | 0.76±0.15 | 0.67±0.20 | 0.69±0.06 | 0.62±0.15 | 0.55±0.13 |  |  |  |
| L-L-M | Colostrum | - | 0.51±0.16 | - | 0.45±0.15 | - | 0.65±0.19 | NS | *** | NS |
|  | 1^st^ month | 0.71±0.18 | 0.57±0.19 | - | 0.42±0.25 | - | 0.68±0.29 |  |  |  |
|  | 4^th^ month | 0.83±0.26 | 0.68±0.24 | 0.47±0.16 | 0.49±0.21 | 0.74±0.32 | 0.61±0.14 |  |  |  |
| S-L-La | Colostrum | - | 0.61±0.21 | - | 0.52±0.22 | - | 0.78±0.26 | NS | * | NS |
|  | 1^st^ month | 0.62±0.09 | 0.60±0.16 | - | 0.67±0.23 | - | 0.72±0.22 |  |  |  |
|  | 4^th^ month | 0.62±0.16 | 0.61±0.15 | 0.54±0.19 | 0.65±0.10 | 0.55±0.18 | 0.61±0.12 |  |  |  |
| L-P-Ca | Colostrum | - | 0.50±0.39 | - | 0.19±0.13 | - | 0.44±0.21 | *** | *** | NS |
|  | 1^st^ month | 0.68±0.23 | 0.66±0.24 | - | 0.58±0.35 | - | 0.62±0.31 |  |  |  |
|  | 4^th^ month | 0.67±0.23 | 0.67±0.26 | 0.42±0.16 | 0.46±0.18 | 0.58±0.26 | 0.45±0.14 |  |  |  |
| S-O-La | Colostrum | - | 0.68±0.20 | - | 0.64±0.27 | - | 0.80±0.27 | *** | NS | NS |
|  | 1^st^ month | 0.55±0.16 | 0.52±0.16 | - | 0.63±0.21 | - | 0.47±0.17 |  |  |  |
|  | 4^th^ month | 0.47±0.12 | 0.47±0.19 | 0.52±0.25 | 0.62±0.16 | 0.42±0.22 | 0.51±0.17 |  |  |  |
| L-L-La | Colostrum | - | 0.31±0.18 | - | 0.20±0.16 | - | 0.39±0.20 | * | *** | * |
|  | 1^st^ month | 0.60±0.22 | 0.47±0.22 | - | 0.30±0.25 | - | 0.69±0.43 |  |  |  |
|  | 4^th^ month | 0.77±0.27 | 0.54±0.25 | 0.30±0.14 | 0.34±0.19 | 0.64±0.33 | 0.48±0.16 |  |  |  |
| S-P-La | Colostrum | - | 0.56±0.27 | - | 0.38±0.24 | - | 0.45±0.23 | NS | NS | NS |
|  | 1^st^ month | 0.57±0.16 | 0.51±0.20 | - | 0.52±0.18 | - | 0.35±0.15 |  |  |  |
|  | 4^th^ month | 0.49±0.17 | 0.49±0.18 | 0.51±0.37 | 0.50±0.18 | 0.43±0.27 | 0.47±0.24 |  |  |  |
| O-L-Ca | Colostrum | - | 0.16±0.10 | - | 0.08±0.08 | - | 0.21±0.11 | *** | *** | * |
|  | 1^st^ month | 0.30±0.11 | 0.25±0.10 | - | 0.16±0.11 | - | 0.32±0.12 |  |  |  |
|  | 4^th^ month | 0.34±0.11 | 0.20±0.07 | 0.12±0.04 | 0.15±0.06 | 0.22±0.08 | 0.20±0.06 |  |  |  |
| O-Po-La | Colostrum | - | 0.21±0.13 | - | 0.11±0.05 | - | 0.21±0.08 | * | * | NS |
|  | 1^st^ month | 0.25±0.06 | 0.24±0.08 | - | 0.22±0.12 | - | 0.23±0.09 |  |  |  |
|  | 4^th^ month | 0.24±0.07 | 0.25±0.08 | 0.18±0.07 | 0.19±0.06 | 0.22±0.09 | 0.19±0.05 |  |  |  |
| L-Po-M | Colostrum | - | 0.16±0.07 | - | 0.12±0.07 | - | 0.19±0.08 | * | *** | NS |
|  | 1^st^ month | 0.22±0.06 | 0.20±0.07 | - | 0.17±0.09 | - | 0.26±0.13 |  |  |  |
|  | 4^th^ month | 0.25±0.08 | 0.21±0.06 | 0.15±0.06 | 0.17±0.07 | 0.23±0.09 | 0.19±0.05 |  |  |  |
| P-P-M | Colostrum | - | 0.14±0.07 | - | 0.09±0.06 | - | 0.11±0.06 | NS | NS | NS |
|  | 1^st^ month | 0.14±0.04 | 0.13±0.05 | - | 0.13±0.05 | - | 0.09±0.04 |  |  |  |
|  | 4^th^ month | 0.12±0.04 | 0.12±0.05 | 0.13±0.09 | 0.13±0.04 | 0.11±0.07 | 0.12±0.06 |  |  |  |
| O-Po-M | Colostrum | - | 0.10±0.04 | - | 0.09±0.04 | - | 0.13±0.04 | NS | * | NS |
|  | 1^st^ month | 0.10±0.02 | 0.10±0.03 | - | 0.11±0.04 | - | 0.12±0.04 |  |  |  |
|  | 4^th^ month | 0.10±0.03 | 0.10±0.03 | 0.09±0.03 | 0.11±0.02 | 0.09±0.03 | 0.10±0.02 |  |  |  |
| P-P-Ca | Colostrum | - | 0.14±0.15 | - | 0.05±0.05 | - | 0.11±0.08 | NS | NS | NS |
|  | 1^st^ month | 0.11±0.03 | 0.12±0.07 | - | 0.13±0.10 | - | 0.09±0.05 |  |  |  |
|  | 4^th^ month | 0.09±0.04 | 0.10±0.05 | 0.09±0.07 | 0.09±0.04 | 0.08±0.06 | 0.10±0.05 |  |  |  |
| Ln-P-La | Colostrum | - | 0.07±0.04 | - | 0.04±0.03 | - | 0.09±0.05 | *** | *** | * |
|  | 1^st^ month | 0.13±0.05 | 0.11±0.04 | - | 0.07±0.05 | - | 0.14±0.05 |  |  |  |
|  | 4^th^ month | 0.15±0.05 | 0.09±0.03 | 0.05±0.02 | 0.06±0.03 | 0.09±0.03 | 0.09±0.03 |  |  |  |
| S-P-Ca | Colostrum | - | 0.11±0.09 | - | 0.05±0.04 | - | 0.09±0.05 | NS | NS | NS |
|  | 1^st^ month | 0.11±0.03 | 0.11±0.05 | - | 0.11±0.05 | - | 0.07±0.03 |  |  |  |
|  | 4^th^ month | 0.09±0.03 | 0.10±0.04 | 0.09±0.07 | 0.09±0.03 | 0.08±0.05 | 0.09±0.04 |  |  |  |
| L-L-Ca | Colostrum | - | 0.03±0.03 | - | 0.01±0.04 | - | 0.05±0.06 | * | *** | *** |
|  | 1^st^ month | 0.15±0.08 | 0.09±0.06 | - | 0.03±0.06 | - | 0.15±0.10 |  |  |  |
|  | 4^th^ month | 0.20±0.10 | 0.07±0.08 | 0.03±0.02 | 0.02±0.03 | 0.11±0.07 | 0.06±0.05 |  |  |  |
| Eo-Po-M | Colostrum | - | 0.08±0.01 | - | 0.09±0.01 | - | 0.09±0.02 | *** | * | NS |
|  | 1^st^ month | 0.06±0.01 | 0.07±0.02 | - | 0.08±0.01 | - | 0.06±0.02 |  |  |  |
|  | 4^th^ month | 0.06±0.01 | 0.06±0.02 | 0.07±0.02 | 0.07±0.01 | 0.05±0.02 | 0.06±0.01 |  |  |  |
| Eo-P-La | Colostrum | - | 0.08±0.02 | - | 0.07±0.03 | - | 0.09±0.03 | ** | NS | NS |
|  | 1^st^ month | 0.06±0.02 | 0.06±0.02 | - | 0.07±0.02 | - | 0.05±0.02 |  |  |  |
|  | 4^th^ month | 0.05±0.01 | 0.05±0.02 | 0.06±0.03 | 0.07±0.02 | 0.05±0.03 | 0.06±0.02 |  |  |  |
| L-Po-La | Colostrum | - | 0.04±0.03 | - | 0.02±0.02 | - | 0.06±0.03 | *** | *** | * |
|  | 1^st^ month | 0.08±0.03 | 0.07±0.03 | - | 0.04±0.03 | - | 0.09±0.03 |  |  |  |
|  | 4^th^ month | 0.09±0.03 | 0.05±0.02 | 0.03±0.01 | 0.04±0.02 | 0.06±0.02 | 0.05±0.02 |  |  |  |
| L-Ln-M | Colostrum | - | 0.02±0.03 | - | 0.02±0.04 | - | 0.06±0.05 | NS | *** | NS |
|  | 1^st^ month | 0.06±0.03 | 0.04±0.04 | - | 0.03±0.04 | - | 0.07±0.07 |  |  |  |
|  | 4^th^ month | 0.11±0.10 | 0.04±0.03 | 0.01±0.03 | 0.03±0.03 | 0.04±0.05 | 0.06±0.05 |  |  |  |
| L-Ln-La | Colostrum | - | 0.00±0.00 | - | 0.01±0.03 | - | 0.03±0.03 | NS | *** | * |
|  | 1^st^ month | 0.04±0.04 | 0.02±0.03 | - | 0.01±0.03 | - | 0.08±0.08 |  |  |  |
|  | 4^th^ month | 0.10±0.13 | 0.02±0.03 | 0.01±0.02 | 0.01±0.02 | 0.03±0.04 | 0.05±0.04 |  |  |  |
| S-S-M | Colostrum | - | 0.04±0.01 | - | 0.04±0.01 | - | 0.02±0.01 | NS | *** | NS |
|  | 1^st^ month | 0.04±0.01 | 0.04±0.01 | - | 0.04±0.02 | - | 0.02±0.01 |  |  |  |
|  | 4^th^ month | 0.04±0.02 | 0.04±0.01 | 0.04±0.02 | 0.04±0.01 | 0.03±0.01 | 0.03±0.01 |  |  |  |
| Ln-P-Ca | Colostrum | - | 0.02±0.03 | - | 0.01±0.02 | - | 0.04±0.04 | NS | *** | * |
|  | 1^st^ month | 0.05±0.04 | 0.03±0.03 | - | 0.02±0.03 | - | 0.05±0.03 |  |  |  |
|  | 4^th^ month | 0.07±0.05 | 0.02±0.02 | 0.01±0.01 | 0.01±0.01 | 0.01±0.02 | 0.03±0.03 |  |  |  |
| Et-P-Ca | Colostrum | - | 0.02±0.01 | - | 0.01±0.01 | - | 0.02±0.01 | ** | *** | * |
|  | 1^st^ month | 0.03±0.01 | 0.03±0.01 | - | 0.02±0.01 | - | 0.03±0.01 |  |  |  |
|  | 4^th^ month | 0.03±0.01 | 0.02±0.01 | 0.01±0.00 | 0.02±0.01 | 0.02±0.01 | 0.02±0.01 |  |  |  |
| O-L-Cy | Colostrum | - | 0.01±0.01 | - | 0.00±0.01 | - | 0.02±0.02 | NS | *** | NS |
|  | 1^st^ month | 0.02±0.02 | 0.01±0.01 | - | 0.01±0.01 | - | 0.02±0.01 |  |  |  |
|  | 4^th^ month | 0.03±0.02 | 0.01±0.01 | 0.00±0.00 | 0.01±0.00 | 0.01±0.01 | 0.01±0.01 |  |  |  |
| ARA-O-La | Colostrum | - | 0.02±0.02 | - | 0.01±0.01 | - | 0.01±0.01 | *** | NS | NS |
|  | 1^st^ month | 0.01±0.01 | 0.01±0.01 | - | 0.01±0.01 | - | 0.01±0.01 |  |  |  |
|  | 4^th^ month | 0.01±0.01 | 0.00±0.00 | 0.00±0.00 | 0.00±0.00 | 0.00±0.01 | 0.00±0.00 |  |  |  |
| MML type |  |  |  |  |  |  |  |  |  |  |
| O-M-La | Colostrum | - | 1.38±0.99 | - | 0.68±0.55 | - | 1.61±0.91 | *** | NS | ** |
|  | 1^st^ month | 1.44±0.39 | 1.54±0.58 | - | 1.98±0.94 | - | 1.67±0.69 |  |  |  |
|  | 4^th^ month | 1.34±0.40 | 1.26±0.49 | 1.03±0.43 | 1.40±0.32 | 1.06±0.49 | 1.20±0.39 |  |  |  |
| O-M-M | Colostrum | - | 0.69±0.35 | - | 0.45±0.28 | - | 0.81±0.31 | NS | NS | ** |
|  | 1^st^ month | 0.65±0.16 | 0.69±0.24 | - | 0.83±0.30 | - | 0.66±0.27 |  |  |  |
|  | 4^th^ month | 0.57±0.15 | 0.62±0.23 | 0.56±0.28 | 0.71±0.12 | 0.51±0.26 | 0.63±0.23 |  |  |  |
| P-M-M | Colostrum | - | 0.53±0.43 | - | 0.25±0.21 | - | 0.42±0.26 | NS | NS | NS |
|  | 1^st^ month | 0.51±0.13 | 0.50±0.23 | - | 0.50±0.23 | - | 0.34±0.16 |  |  |  |
|  | 4^th^ month | 0.42±0.14 | 0.45±0.19 | 0.43±0.32 | 0.42±0.14 | 0.37±0.24 | 0.42±0.21 |  |  |  |
| O-La-La | Colostrum | - | 0.47±0.43 | - | 0.19±0.17 | - | 0.53±0.42 | ** | NS | ** |
|  | 1^st^ month | 0.46±0.20 | 0.47±0.25 | - | 0.60±0.39 | - | 0.60±0.31 |  |  |  |
|  | 4^th^ month | 0.47±0.19 | 0.36±0.14 | 0.25±0.12 | 0.39±0.10 | 0.30±0.15 | 0.35±0.12 |  |  |  |
| P-M-La | Colostrum | - | 0.52±0.56 | - | 0.18±0.19 | - | 0.41±0.31 | NS | NS | NS |
|  | 1^st^ month | 0.43±0.12 | 0.44±0.25 | - | 0.50±0.37 | - | 0.34±0.18 |  |  |  |
|  | 4^th^ month | 0.35±0.14 | 0.39±0.20 | 0.32±0.26 | 0.35±0.13 | 0.31±0.22 | 0.37±0.20 |  |  |  |
| L-M-M | Colostrum | - | 0.32±0.19 | - | 0.16±0.08 | - | 0.31±0.12 | * | * | NS |
|  | 1^st^ month | 0.37±0.09 | 0.36±0.12 | - | 0.33±0.17 | - | 0.35±0.13 |  |  |  |
|  | 4^th^ month | 0.35±0.10 | 0.37±0.12 | 0.27±0.10 | 0.29±0.08 | 0.33±0.14 | 0.28±0.07 |  |  |  |
| S-M-La | Colostrum | - | 0.38±0.31 | - | 0.18±0.15 | - | 0.30±0.18 | NS | NS | NS |
|  | 1^st^ month | 0.36±0.09 | 0.35±0.16 | - | 0.35±0.16 | - | 0.24±0.11 |  |  |  |
|  | 4^th^ month | 0.30±0.10 | 0.32±0.13 | 0.30±0.23 | 0.30±0.10 | 0.26±0.17 | 0.29±0.15 |  |  |  |
| L-M-La | Colostrum | - | 0.26±0.21 | - | 0.10±0.07 | - | 0.23±0.11 | *** | *** | NS |
|  | 1^st^ month | 0.36±0.12 | 0.35±0.13 | - | 0.31±0.18 | - | 0.33±0.17 |  |  |  |
|  | 4^th^ month | 0.36±0.12 | 0.35±0.14 | 0.22±0.08 | 0.25±0.09 | 0.31±0.14 | 0.24±0.07 |  |  |  |
| L-La-La | Colostrum | - | 0.21±0.22 | - | 0.06±0.07 | - | 0.17±0.11 | * | *** | NS |
|  | 1^st^ month | 0.29±0.15 | 0.26±0.15 | - | 0.23±0.18 | - | 0.29±0.20 |  |  |  |
|  | 4^th^ month | 0.32±0.14 | 0.26±0.13 | 0.13±0.06 | 0.18±0.09 | 0.22±0.12 | 0.17±0.07 |  |  |  |
| O-M-Ca | Colostrum | - | 0.24±0.22 | - | 0.10±0.09 | - | 0.28±0.22 | ** | NS | ** |
|  | 1^st^ month | 0.24±0.10 | 0.24±0.13 | - | 0.31±0.20 | - | 0.31±0.16 |  |  |  |
|  | 4^th^ month | 0.24±0.10 | 0.19±0.07 | 0.13±0.06 | 0.20±0.05 | 0.15±0.08 | 0.18±0.06 |  |  |  |
| O-La-Ca | Colostrum | - | 0.19±0.19 | - | 0.06±0.09 | - | 0.22±0.21 | *** | *** | ** |
|  | 1^st^ month | 0.25±0.15 | 0.23±0.14 | - | 0.24±0.16 | - | 0.30±0.17 |  |  |  |
|  | 4^th^ month | 0.27±0.13 | 0.15±0.07 | 0.08±0.05 | 0.16±0.07 | 0.12±0.06 | 0.12±0.06 |  |  |  |
| P-M-Ca | Colostrum | - | 0.21±0.27 | - | 0.05±0.06 | - | 0.16±0.13 | NS | NS | * |
|  | 1^st^ month | 0.15±0.06 | 0.16±0.11 | - | 0.20±0.19 | - | 0.14±0.09 |  |  |  |
|  | 4^th^ month | 0.13±0.06 | 0.14±0.08 | 0.09±0.08 | 0.12±0.05 | 0.11±0.08 | 0.13±0.08 |  |  |  |
| S-M-M | Colostrum | - | 0.14±0.07 | - | 0.09±0.06 | - | 0.11±0.06 | NS | NS | NS |
|  | 1^st^ month | 0.14±0.04 | 0.13±0.05 | - | 0.13±0.05 | - | 0.09±0.04 |  |  |  |
|  | 4^th^ month | 0.12±0.04 | 0.12±0.05 | 0.13±0.09 | 0.13±0.04 | 0.11±0.07 | 0.12±0.06 |  |  |  |
| Po-M-La | Colostrum | - | 0.12±0.11 | - | 0.05±0.04 | - | 0.13±0.11 | ** | NS | ** |
|  | 1^st^ month | 0.11±0.05 | 0.12±0.06 | - | 0.15±0.10 | - | 0.15±0.08 |  |  |  |
|  | 4^th^ month | 0.12±0.05 | 0.09±0.04 | 0.06±0.03 | 0.10±0.03 | 0.07±0.04 | 0.09±0.03 |  |  |  |
| L-M-Ca | Colostrum | - | 0.08±0.08 | - | 0.02±0.03 | - | 0.07±0.04 | ** | *** | NS |
|  | 1^st^ month | 0.11±0.06 | 0.10±0.06 | - | 0.09±0.07 | - | 0.11±0.08 |  |  |  |
|  | 4^th^ month | 0.12±0.05 | 0.10±0.05 | 0.05±0.02 | 0.07±0.04 | 0.09±0.05 | 0.07±0.03 |  |  |  |
| L-La-Ca | Colostrum | - | 0.08±0.09 | - | 0.02±0.04 | - | 0.06±0.06 | * | *** | NS |
|  | 1^st^ month | 0.15±0.11 | 0.11±0.08 | - | 0.08±0.07 | - | 0.12±0.10 |  |  |  |
|  | 4^th^ month | 0.17±0.10 | 0.09±0.07 | 0.03±0.02 | 0.05±0.05 | 0.07±0.05 | 0.04±0.03 |  |  |  |
| S-La-La | Colostrum | - | 0.12±0.13 | - | 0.04±0.04 | - | 0.09±0.07 | NS | NS | NS |
|  | 1^st^ month | 0.10±0.03 | 0.10±0.06 | - | 0.11±0.08 | - | 0.08±0.04 |  |  |  |
|  | 4^th^ month | 0.08±0.03 | 0.09±0.05 | 0.07±0.06 | 0.08±0.03 | 0.07±0.05 | 0.08±0.05 |  |  |  |
| S-M-Ca | Colostrum | - | 0.11±0.12 | - | 0.04±0.04 | - | 0.09±0.07 | NS | NS | NS |
|  | 1^st^ month | 0.09±0.03 | 0.09±0.05 | - | 0.11±0.08 | - | 0.07±0.04 |  |  |  |
|  | 4^th^ month | 0.08±0.03 | 0.08±0.04 | 0.07±0.06 | 0.08±0.03 | 0.07±0.05 | 0.08±0.04 |  |  |  |
| S-La-Ca | Colostrum | - | 0.10±0.14 | - | 0.03±0.03 | - | 0.08±0.06 | NS | NS | * |
|  | 1^st^ month | 0.07±0.03 | 0.08±0.05 | - | 0.10±0.09 | - | 0.07±0.05 |  |  |  |
|  | 4^th^ month | 0.06±0.03 | 0.07±0.04 | 0.05±0.04 | 0.06±0.03 | 0.05±0.04 | 0.06±0.04 |  |  |  |
| P-La-Ca | Colostrum | - | 0.11±0.14 | - | 0.00±0.00 | - | 0.02±0.03 | NS | *** | NS |
|  | 1^st^ month | 0.08±0.04 | 0.08±0.06 | - | 0.00±0.00 | - | 0.04±0.07 |  |  |  |
|  | 4^th^ month | 0.07±0.03 | 0.07±0.04 | 0.04±0.03 | 0.00±0.00 | 0.05±0.03 | 0.02±0.04 |  |  |  |
| Ed-M-M | Colostrum | - | 0.04±0.01 | - | 0.03±0.01 | - | 0.05±0.02 | NS | * | NS |
|  | 1^st^ month | 0.04±0.01 | 0.04±0.01 | - | 0.04±0.01 | - | 0.04±0.01 |  |  |  |
|  | 4^th^ month | 0.04±0.01 | 0.04±0.01 | 0.03±0.01 | 0.04±0.01 | 0.03±0.01 | 0.04±0.01 |  |  |  |
| Ln-M-M | Colostrum | - | 0.03±0.02 | - | 0.01±0.01 | - | 0.03±0.02 | ** | *** | * |
|  | 1^st^ month | 0.05±0.02 | 0.04±0.02 | - | 0.03±0.02 | - | 0.05±0.02 |  |  |  |
|  | 4^th^ month | 0.05±0.02 | 0.03±0.01 | 0.02±0.01 | 0.02±0.01 | 0.04±0.01 | 0.03±0.01 |  |  |  |
| Po-M-Ca | Colostrum | - | 0.02±0.02 | - | 0.01±0.01 | - | 0.02±0.02 | *** | ** | ** |
|  | 1^st^ month | 0.03±0.02 | 0.03±0.02 | - | 0.03±0.02 | - | 0.03±0.02 |  |  |  |
|  | 4^th^ month | 0.03±0.01 | 0.02±0.01 | 0.01±0.01 | 0.02±0.01 | 0.01±0.01 | 0.01±0.01 |  |  |  |
| Ln-M-La | Colostrum | - | 0.01±0.01 | - | 0.00±0.01 | - | 0.02±0.02 | NS | *** | * |
|  | 1^st^ month | 0.03±0.02 | 0.02±0.02 | - | 0.01±0.01 | - | 0.03±0.02 |  |  |  |
|  | 4^th^ month | 0.04±0.03 | 0.01±0.01 | 0.00±0.00 | 0.01±0.01 | 0.01±0.01 | 0.01±0.01 |  |  |  |
| Total MLCT | Colostrum | - | 29.41±12.17 | - | 21.59±7.74 | - | 32.13±9.19 | NS | NS | NS |
|  | 1^st^ month | 29.90±5.04 | 29.28±7.66 | - | 30.65±10.19 | - | 29.20±9.21 |  |  |  |
|  | 4^th^ month | 28.82±5.99 | 27.29±6.97 | 23.55±8.27 | 27.34±4.10 | 24.72±8.65 | 25.20±5.89 |  |  |  |

NS, *P*>0.05; *, *P*<0.05; **, *P*<0.01; ***, *P*<0.001. Co, 6:0; Cy, 8:0; Ca, 10:0; La, 12:0; M, 14:0; Pa, 15:0; P, 16:0; Po, 16:1; Ha, 17:0; He, 17:1; O, 18:1; S, 18:0; L, 18:2; Ln, 18:3; No, 19:1; E, 20:0; Eo, 20:1; Ed, 20:2; Et, 20:3; ARA, 20:4; Do, 22:0.

**Table S3.** Composition of UPU (wt %) in human milk at different lactation periods and regions

| TAGs | Lactational  periods | Baotou | Beijing | Jinan | Kunming | Shenzhen | Xining | P1 (lactations) | P2 (regions) | P1*P2 |
| --- | --- | --- | --- | --- | --- | --- | --- | --- | --- | --- |
| O-P-L | Colostrum | - | 16.16±2.80 | - | 15.94±2.17 | - | 14.45±2.90 | NS | *** | NS |
|  | 1^st^ month | 16.10±1.49 | 16.92±2.09 | - | 15.30±2.38 | - | 14.55±1.96 |  |  |  |
|  | 4^th^ month | 14.63±2.16 | 17.91±2.00 | 17.49±1.98 | 15.63±1.76 | 17.25±1.82 | 15.14±1.32 |  |  |  |
| O-P-O | Colostrum | - | 14.41±3.11 | - | 15.50±1.94 | - | 13.61±2.68 | NS | *** | NS |
|  | 1^st^ month | 12.90±1.40 | 14.00±2.20 | - | 15.40±3.33 | - | 13.06±2.65 |  |  |  |
|  | 4^th^ month | 12.01±1.95 | 13.80±2.05 | 15.77±2.27 | 15.49±1.57 | 12.89±2.20 | 14.18±2.00 |  |  |  |
| L-L-P | Colostrum | - | 3.97±0.61 | - | 3.88±0.97 | - | 3.93±1.11 | ** | *** | NS |
|  | 1^st^ month | 5.64±1.18 | 5.72±1.68 | - | 3.70±1.66 | - | 4.64±1.17 |  |  |  |
|  | 4^th^ month | 6.36±1.49 | 6.46±1.52 | 4.98±1.38 | 4.12±1.34 | 6.85±2.01 | 4.88±1.42 |  |  |  |
| Et-L-P | Colostrum | - | 0.73±0.18 | - | 0.98±0.35 | - | 0.83±0.23 | NS | *** | * |
|  | 1^st^ month | 1.16±0.39 | 0.95±0.36 | - | 0.52±0.39 | - | 1.40±0.80 |  |  |  |
|  | 4^th^ month | 1.45±0.40 | 1.10±0.70 | 0.91±0.46 | 0.63±0.29 | 1.81±0.83 | 1.37±0.71 |  |  |  |
| O-P-Po | Colostrum | - | 1.20±0.20 | - | 1.26±0.16 | - | 1.38±0.27 | *** | * | NS |
|  | 1^st^ month | 0.93±0.21 | 1.01±0.25 | - | 1.11±0.20 | - | 0.92±0.30 |  |  |  |
|  | 4^th^ month | 0.89±0.16 | 0.81±0.24 | 0.97±0.27 | 1.06±0.10 | 0.77±0.22 | 0.83±0.15 |  |  |  |
| Ed-O-P | Colostrum | - | 0.48±0.19 | - | 0.71±0.17 | - | 0.53±0.15 | ** | *** | NS |
|  | 1^st^ month | 0.37±0.08 | 0.35±0.07 | - | 0.45±0.14 | - | 0.57±0.24 |  |  |  |
|  | 4^th^ month | 0.43±0.10 | 0.39±0.10 | 0.52±0.21 | 0.53±0.17 | 0.45±0.15 | 0.60±0.18 |  |  |  |
| L-Ln-P | Colostrum | - | 0.33±0.07 | - | 0.32±0.11 | - | 0.43±0.13 | NS | *** | NS |
|  | 1^st^ month | 0.55±0.17 | 0.51±0.18 | - | 0.29±0.20 | - | 0.56±0.16 |  |  |  |
|  | 4^th^ month | 0.77±0.48 | 0.46±0.16 | 0.34±0.16 | 0.32±0.14 | 0.47±0.19 | 0.54±0.27 |  |  |  |
| ARA-O-P | Colostrum | - | 0.18±0.04 | - | 0.24±0.08 | - | 0.20±0.06 | NS | *** | * |
|  | 1^st^ month | 0.28±0.09 | 0.23±0.09 | - | 0.12±0.09 | - | 0.34±0.19 |  |  |  |
|  | 4^th^ month | 0.35±0.10 | 0.26±0.17 | 0.22±0.11 | 0.15±0.07 | 0.43±0.20 | 0.33±0.17 |  |  |  |
| Et-O-P | Colostrum | - | 0.13±0.04 | - | 0.19±0.04 | - | 0.15±0.04 | NS | ** | ** |
|  | 1^st^ month | 0.14±0.03 | 0.13±0.03 | - | 0.10±0.04 | - | 0.18±0.07 |  |  |  |
|  | 4^th^ month | 0.15±0.04 | 0.14±0.06 | 0.14±0.05 | 0.12±0.04 | 0.18±0.06 | 0.18±0.06 |  |  |  |
| Eo-L-P | Colostrum | - | 0.08±0.03 | - | 0.11±0.03 | - | 0.08±0.02 | ** | *** | NS |
|  | 1^st^ month | 0.06±0.01 | 0.06±0.01 | - | 0.07±0.02 | - | 0.09±0.04 |  |  |  |
|  | 4^th^ month | 0.07±0.02 | 0.06±0.02 | 0.08±0.03 | 0.08±0.03 | 0.07±0.02 | 0.09±0.03 |  |  |  |
| O-He-P | Colostrum | - | 0.07±0.01 | - | 0.07±0.02 | - | 0.08±0.03 | NS | ** | NS |
|  | 1^st^ month | 0.09±0.03 | 0.08±0.03 | - | 0.07±0.02 | - | 0.06±0.04 |  |  |  |
|  | 4^th^ month | 0.09±0.06 | 0.06±0.03 | 0.07±0.04 | 0.08±0.02 | 0.05±0.03 | 0.06±0.04 |  |  |  |
| L-He-P | Colostrum | - | 0.02±0.00 | - | 0.02±0.01 | - | 0.03±0.01 | NS | *** | NS |
|  | 1^st^ month | 0.04±0.01 | 0.03±0.01 | - | 0.02±0.01 | - | 0.02±0.01 |  |  |  |
|  | 4^th^ month | 0.04±0.01 | 0.03±0.01 | 0.02±0.01 | 0.02±0.01 | 0.02±0.01 | 0.02±0.02 |  |  |  |
| No-O-P | Colostrum | - | 0.02±0.01 | - | 0.03±0.01 | - | 0.03±0.01 | NS | ** | NS |
|  | 1^st^ month | 0.02±0.01 | 0.02±0.01 | - | 0.02±0.01 | - | 0.02±0.02 |  |  |  |
|  | 4^th^ month | 0.04±0.04 | 0.01±0.01 | 0.02±0.02 | 0.03±0.01 | 0.01±0.01 | 0.03±0.02 |  |  |  |
| Eo-O-P | Colostrum | - | 0.02±0.01 | - | 0.03±0.01 | - | 0.02±0.00 | NS | *** | NS |
|  | 1^st^ month | 0.01±0.00 | 0.01±0.00 | - | 0.02±0.01 | - | 0.02±0.01 |  |  |  |
|  | 4^th^ month | 0.02±0.01 | 0.01±0.00 | 0.02±0.01 | 0.02±0.01 | 0.02±0.01 | 0.02±0.01 |  |  |  |
| No-L-P | Colostrum | - | 0.01±0.01 | - | 0.02±0.01 | - | 0.02±0.01 | NS | ** | NS |
|  | 1^st^ month | 0.02±0.01 | 0.02±0.01 | - | 0.01±0.00 | - | 0.02±0.01 |  |  |  |
|  | 4^th^ month | 0.03±0.02 | 0.02±0.01 | 0.02±0.01 | 0.02±0.00 | 0.02±0.01 | 0.02±0.01 |  |  |  |
| Total UPU | Colostrum | - | 37.81±6.75 | - | 39.30±4.49 | - | 35.78±6.02 | NS | ** | NS |
|  | 1^st^ month | 38.32±2.76 | 40.03±4.81 | - | 37.20±5.46 | - | 36.44±4.15 |  |  |  |
|  | 4^th^ month | 37.30±3.59 | 41.51±3.96 | 41.59±4.47 | 38.31±2.84 | 41.30±4.51 | 38.29±2.95 |  |  |  |

NS, *P*>0.05; *, *P*<0.05; **, *P*<0.01; ***, *P*<0.001. P, 16:0; Po, 16:1; He, 17:1; O, 18:1; L, 18:2; Ln, 18:3; No, 19:1; Eo, 20:1; Ed, 20:2; Et, 20:3; ARA, 20:4.

Figure S1. OPLS-DA analysis of TAG species between boys and girls.


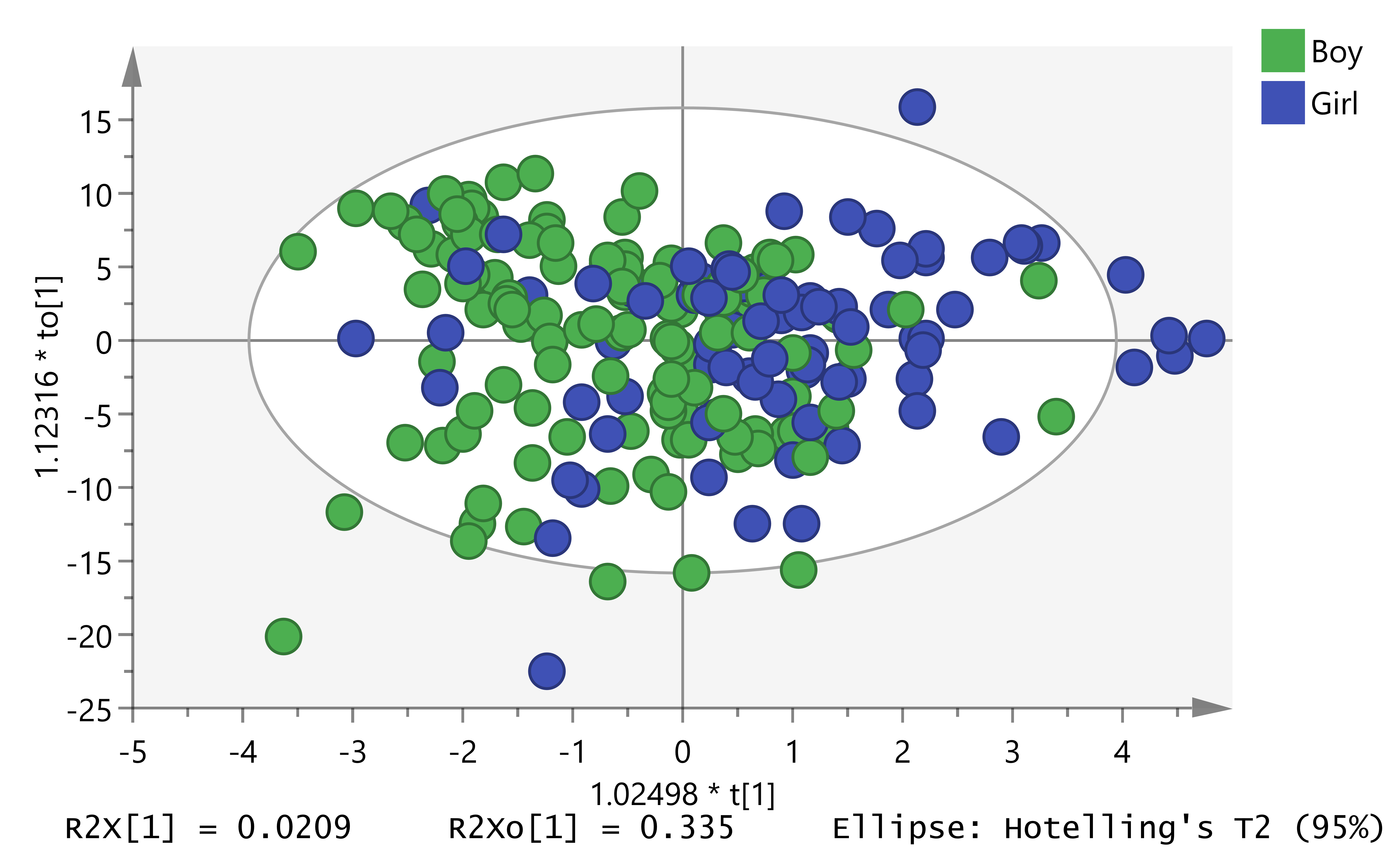

Supplement: Supplementary file 1 [file Data_Sheet_1.docx]
